# Squalene-Tetrahymanol Cyclase Expression Enables Sterol-Independent Growth of Saccharomyces cerevisiae

**DOI:** 10.1128/AEM.00672-20

**Published:** 2020-08-18

**Authors:** Sanne J. Wiersma, Christiaan Mooiman, Martin Giera, Jack T. Pronk

**Affiliations:** a Department of Biotechnology, Delft University of Technology, Delft, The Netherlands; b Center for Proteomics and Metabolomics, Leiden University Medical Center, Leiden, The Netherlands; Nanjing Agricultural University

**Keywords:** *Saccharomyces cerevisiae*, anaerobic, membrane composition, oxygen requirements, sterols, tetrahymanol

## Abstract

Biosynthesis of sterols, which are considered essential components of virtually all eukaryotic membranes, requires molecular oxygen. Anaerobic growth of the yeast Saccharomyces cerevisiae therefore strictly depends on sterol supplementation of synthetic growth media. Neocallimastigomycota are a group of strictly anaerobic fungi which, instead of containing sterols, contain the pentacyclic triterpenoid “sterol surrogate” tetrahymanol, which is formed by cyclization of squalene. Here, we demonstrate that expression of the squalene-tetrahymanol cyclase gene *TtTHC1* from the ciliate Tetrahymena thermophila enables synthesis of tetrahymanol by S. cerevisiae. Moreover, expression of *TtTHC1* enabled exponential growth of anaerobic S. cerevisiae cultures in sterol-free synthetic media. After deletion of the *ERG1* gene from a *TtTHC1*-expressing S. cerevisiae strain, native sterol synthesis was abolished and sustained sterol-free growth was demonstrated under anaerobic as well as aerobic conditions. Anaerobic cultures of *TtTHC1*-expressing S. cerevisiae on sterol-free medium showed lower specific growth rates and biomass yields than ergosterol-supplemented cultures, while their ethanol yield was higher. This study demonstrated that acquisition of a functional squalene-tetrahymanol cyclase gene offers an immediate growth advantage to S. cerevisiae under anaerobic, sterol-limited conditions and provides the basis for a metabolic engineering strategy to eliminate the oxygen requirements associated with sterol synthesis in yeasts.

**IMPORTANCE** The laboratory experiments described in this report simulate a proposed horizontal gene transfer event during the evolution of strictly anaerobic fungi. The demonstration that expression of a single heterologous gene sufficed to eliminate anaerobic sterol requirements in the model eukaryote Saccharomyces cerevisiae therefore contributes to our understanding of how sterol-independent eukaryotes evolved in anoxic environments. This report provides a proof of principle for a metabolic engineering strategy to eliminate sterol requirements in yeast strains that are applied in large-scale anaerobic industrial processes. The sterol-independent yeast strains described in this report provide a valuable platform for further studies on the physiological roles and impacts of sterols and sterol surrogates in eukaryotic cells.

## Abstract (precis)

The laboratory experiments described in this report simulate a proposed horizontal gene transfer event during the evolution of strictly anaerobic fungi. The demonstration that expression of a single heterologous gene sufficed to eliminate anaerobic sterol requirements in the model eukaryote Saccharomyces cerevisiae therefore contributes to our understanding of how sterol-independent eukaryotes evolved in anoxic environments. This report provides a proof of principle for a metabolic engineering strategy to eliminate sterol requirements in yeast strains that are applied in large-scale anaerobic industrial processes. The sterol-independent yeast strains described in this report provide a valuable platform for further studies on the physiological roles and impacts of sterols and sterol surrogates in eukaryotic cells.

## INTRODUCTION

Sterols are a class of hydrophobic triterpenoid compounds, representatives of which are found in almost all eukaryotic membranes. Sterols affect membrane fluidity and permeability (1, 2), and sterol-enriched domains (“lipid rafts”) contribute to lateral compartmentalization of eukaryotic plasma membranes by influencing localization of specific membrane proteins (3, 4). Cholesterol is the major sterol in mammals, phytosterol the major sterol in plants, and ergosterol the major sterol in filamentous fungi and yeasts (5).

Saccharomyces cerevisiae is an intensively used model for studying sterol function and biosynthesis in eukaryotes. Analysis of sterol-synthesis mutants of this yeast has revealed a wide range of cellular processes that are influenced by sterol composition (reviewed in references 6 and 7). These include endocytosis (8), intracellular trafficking and excretion of proteins (9), and nutrient uptake (10). In addition, sterols influence resistance to stresses, such as superoptimal temperature and presence of growth-inhibiting compounds (11–13). Based on the importance of sterols for fungal growth, many fungicides target ergosterol biosynthesis (14–16).

**FIG 1:**
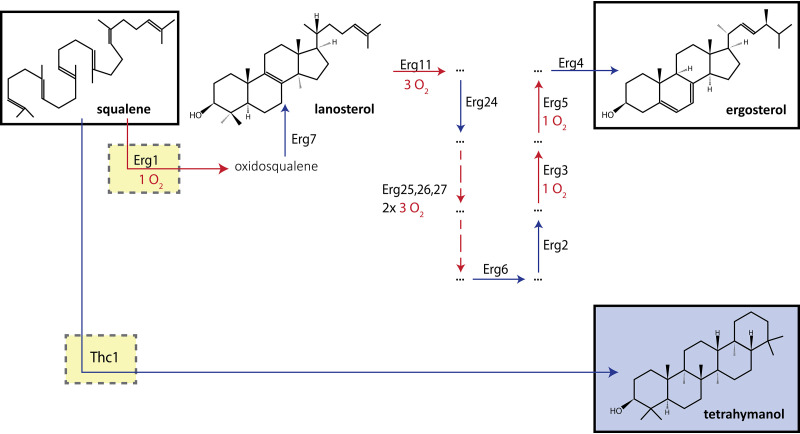
Schematic overview of ergosterol and tetrahymanol synthesis pathways. Erg1, Erg7, Erg24, Erg25, Erg26, Erg27, Erg6, Erg2, Erg3, Erg5, and Erg4, native S. cerevisiae enzymes involved in the oxygen-dependent synthesis of ergosterol from squalene. Thc1, squalene tetrahymanol cyclase from Tetrahymena thermophila. Oxidosqualene, epoxidation product of squalene, formed by Erg1. Lanosterol, first tetracyclic compound in fungal ergosterol synthesis. For oxygen-dependent reactions, the number of moles of oxygen required is indicated. Dashed boxes represent enzymes whose expression levels were modified in this study.

Eukaryotic sterol synthesis starts with the oxygen-independent conversion of acetyl coenzyme A (acetyl-CoA) into squalene via the mevalonate pathway or, in plant plastids, via the 2-*C*-methyl-d-erythritol 4-phosphate pathway (17). The subsequent conversion of squalene into specific sterols involves a strongly conserved oxygen-dependent pathway (18), whose activity is initiated by the oxygen-dependent epoxidation of squalene to oxidosqualene, a reaction catalyzed by Erg1 in S. cerevisiae. Cyclization of oxidosqualene to lanosterol, which yields the basic tetracyclic sterol backbone structure, is followed by a series of further modifications, several of which require molecular oxygen. The complete synthesis of a single molecule of ergosterol from squalene requires 12 molecules of oxygen (Fig. 1).

No evidence is available for anaerobic sterol biosynthesis in either living organisms or the geological record (5). Consistent with the oxygen dependency of sterol biosynthesis, early yeast research already demonstrated that S. cerevisiae is strictly auxotrophic for sterols under anaerobic conditions (19). S. cerevisiae transporters Pdr11 and Aus1, which mediate ATP-dependent import of sterols across the plasma membrane, are expressed only at very low oxygen concentrations (20, 21). While ergosterol is routinely included in synthetic media (SM) for anaerobic growth of S. cerevisiae, several other sterols can also complement the sterol auxotrophy of anaerobic cultures and heme-incompetent cells. However, several studies previously reported that sterols with specific structural features are required to successfully complete the yeast cell cycle (22–24).

Together with the synthesis of unsaturated fatty acids, which in eukaryotes also requires oxygen (25, 26), the oxygen requirement for sterol synthesis strongly affects large-scale, anaerobic industrial applications of *Saccharomyces* yeasts. Anaerobic wine and beer fermentation processes are commonly preceded by a brief aeration phase that enables yeast cells to synthesize and store sterols and unsaturated fatty acids (27, 28). Preliminary arrest of the subsequent anaerobic phase of these processes (“stuck fermentation”) is often attributed to premature depletion of the remaining lipid reserves (29, 30).

Neocallimastigomycota, a group of obligately anaerobic rumen fungi, lack the genetic information for sterol biosynthesis (31–33). Instead, their membranes contain tetrahymanol, a pentacyclic triterpenoid compound that is considered to act as a sterol surrogate (34–36) and whose synthesis by Neocallimastigomycota is seen as a key evolutionary adaptation to their anaerobic lifestyle (37, 38). In contrast to the multistep, oxygen-dependent synthesis of sterols from squalene, tetrahymanol can be produced from this intermediate in a single, oxygen-independent cyclization reaction catalyzed by squalene-tetrahymanol cyclase (STC; EC 4.2.1.123) (39).

Originally discovered in the protozoan Tetrahymena pyriformis (40), tetrahymanol also occurs in the fern Oleandra wallichii (41) and in several prokaryotes (42–44). Indeed, DNA sequence analysis indicated that Neocallimastigomycota acquired a prokaryotic STC gene by horizontal gene transfer (32). However, whether the mere acquisition of a functional STC gene is sufficient to reduce or even eliminate sterol requirements of fungi has not yet been investigated. Addressing this issue may provide not only insight into the roles of sterols and sterol surrogates in eukaryotes and in the evolution of an anaerobic lifestyle in eukaryotes but also strategies to reduce or eliminate oxygen requirements in anaerobic applications of yeasts and other fungi.

The goals of the present study were to analyze the impact of expression of the STC gene of T. thermophila in wild-type and sterol biosynthesis-deficient S. cerevisiae strains on triterpenoid and fatty acid composition as well as on sterol requirements and growth rates in anaerobic and aerobic cultures.

## RESULTS

### Expression of a Tetrahymena thermophila squalene-tetrahymanol cyclase gene enables tetrahymanol synthesis in S. cerevisiae.

**FIG 2:**
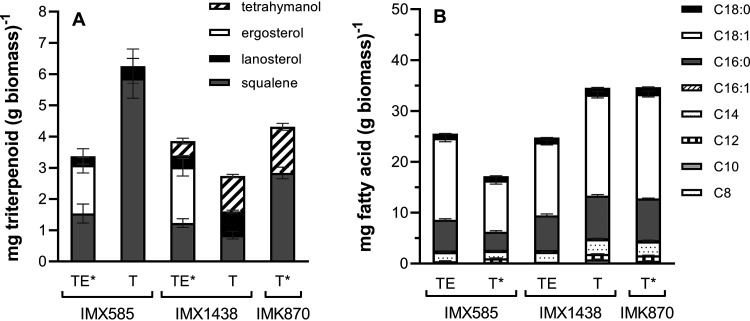
Analysis of triterpenoid fraction and total fatty acid composition of anaerobic biomass of S. cerevisiae strains IMX585 and IMX1438. Biomass was harvested in the second anaerobic sequential batch reactor (SBR) cultivation cycle after the initial cycle for depletion of anaerobic growth factors. For the IMX585 reference strain, biomass was harvested at the end of this SBR cycle. For IMX1438 (*sga1*Δ::*TtTHC1*) and IMK870 (*sga1*Δ::*TtTHC1 erg1*Δ), biomass was harvested during exponential growth. Unless otherwise indicated, data represent means and standard error of the means of results from three replicate SBR experiments. (A) Triterpenoid fraction. (B) Fatty acid composition of biomass harvested during anaerobic sequential batch reactor experiments. TE, culture supplemented with both Tween 80 and ergosterol; T, culture supplemented with Tween 80 only; *, data represent results of two replicate SBR experiments.

**FIG 3:**
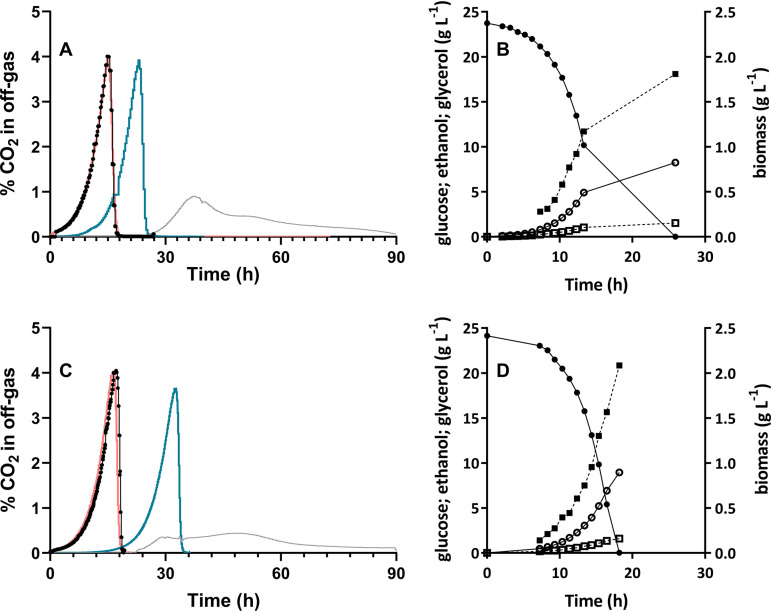
Anaerobic sequential batch bioreactor (SBR) cultivation of S. cerevisiae strains IMX585 and IMX1438 with sterol supplementation. All panels represent data from a single representative SBR experiment performed at 30°C on SMD-urea without pH control. Data from replicate experiments are shown in Fig. S4. (A and B) Reference strain IMX585. (C and D) Strain IMX1438 (*sga1*Δ::*TtTHC1*). (A and C) Percentages of CO_2_ in off-gas during the initial batch cycle without anaerobic growth factors (gray line) and during the first (blue line), second (black line and dots), and third (red line) subsequent SBR cycles on SMD-urea supplemented with Tween 80 and ergosterol. (B and D) Concentrations of glucose (closed circles), biomass (closed squares), ethanol (open circles), and glycerol (open squares).

The squalene-tetrahymanol cyclase (STC) gene *TtTHC1* of Tetrahymena thermophila was codon optimized for expression in S. cerevisiae and integrated into the genome of Cas9-expressing reference strain IMX585 (45) under the control of the constitutive *TEF1* promoter. To investigate the impact of *TtTHC1* expression under anaerobic, sterol-sufficient conditions, growth of the resulting strain, IMX1438 (*sga1*Δ::*TtTHC1*), was compared to that of the IMX585 reference strain in anaerobic sequential batch reactor (SBR) cultures grown on SMD-urea (synthetic media supplemented with glucose and urea) supplemented with Tween 80 and ergosterol. Tetrahymanol was detected in cultures of strain IMX1438 (*sga1*Δ::*TtTHC1*) at cellular contents of 0.47 ± 0.09 mg (g biomass)^−1^ whereas, as anticipated, no tetrahymanol was found in the IMX585 reference strain (Fig. 2A; see also Fig. S2 and Table S2 in the supplemental material). Neither the cellular levels of squalene, lanosterol, and ergosterol nor the fatty acid levels and compositions of the two strains showed marked differences under these conditions (Fig. 2; see also Table S2 and S3). Strains IMX585 and IMX1438 (*sga1*Δ::*TtTHC1*) both exhibited fast exponential growth in three subsequent anaerobic SBR cycles on ergosterol-supplemented SMD-urea (Fig. 3A and C; see also Fig. S4), with glucose being consumed within 26 h (Fig. 3B and D; see also Fig. S4). The specific growth rates and biomass yields of the two strains differed by less than 10% (Table 1), and no major differences were observed between their extracellular metabolite profiles (Fig. 3B and D; see also Table S1). These results indicated that tetrahymanol production by strain IMX1438 did not have a major impact on its physiology in anaerobic ergosterol-supplemented cultures.

**TABLE 1:** Specific growth rates and biomass yields of S. cerevisiae strains in anaerobic sequential batch reactor experiments^*a*^

*S. cerevisiae* strain	Relevant genotype	Sterol supplementation	Growth rate (h^−1^)	Biomass yield (g biomass [g glucose]^−1^)	Ethanol yield (g ethanol [g glucose]^−1^)
IMX585	*ERG1* (reference strain)	Yes	0.26 ± 0.00	0.086 ± 0.001	0.37 ± 0.00
		No^*b*^^,^^*c*^	∼0.034 ± 0.000	∼0.027 ± 0.002	∼0.38 ± 0.01

IMX1438	*sga1*Δ::*TtTHC1*	Yes	0.24 ± 0.01 (A)	0.082 ± 0.000 (B)	0.37 ± 0.00 (C)
		No	0.15 ± 0.01 (A)	0.052 ± 0.000 (B)	0.39 ± 0.00 (C)

IMK870	*sga1*Δ::*TtTHC1 erg1*Δ	No^*b*^	0.11 ± 0.01	0.049 ± 0.006	0.39 ± 0.02

a Strains were grown on glucose synthetic medium (SMD-urea) with Tween 80, with or without supplementation with ergosterol, at 30°C. Unless otherwise indicated, data are represented as averages ± standard errors of measurements of results from three independent bioreactor experiments. Levels of recovery of glucose carbon in biomass, CO_2_, and soluble organic products were between 95% and 105% for all experiments. Growth rate and biomass yield data labeled with matching uppercase letters in parentheses indicate pairs of data sets for which the means were determined to be significantly different using a two-tailed unpaired Student's *t* test with a *P* value of 0.05.

b Data represent results obtained with two replicates.

c Nonexponential growth; estimate based on measurements at start and end of SBR cycles.

### Tetrahymanol synthesis supports anaerobic growth in the absence of sterol supplementation.

To investigate whether tetrahymanol can functionally replace ergosterol in anaerobic cultures of S. cerevisiae, strains IMX1438 (*sga1*Δ::*TtTHC1*) and IMX585 were tested in anaerobic SBR cultures on SMD-urea without ergosterol. SBR experiments were preceded by a single batch-cultivation cycle on SMD-urea with neither ergosterol nor Tween 80, included to deplete endogenous reserves of sterols and unsaturated fatty acids. This initial culture was followed by three SBR cycles on SMD-urea supplemented with Tween 80 but lacking a source of sterol.

**FIG 4:**
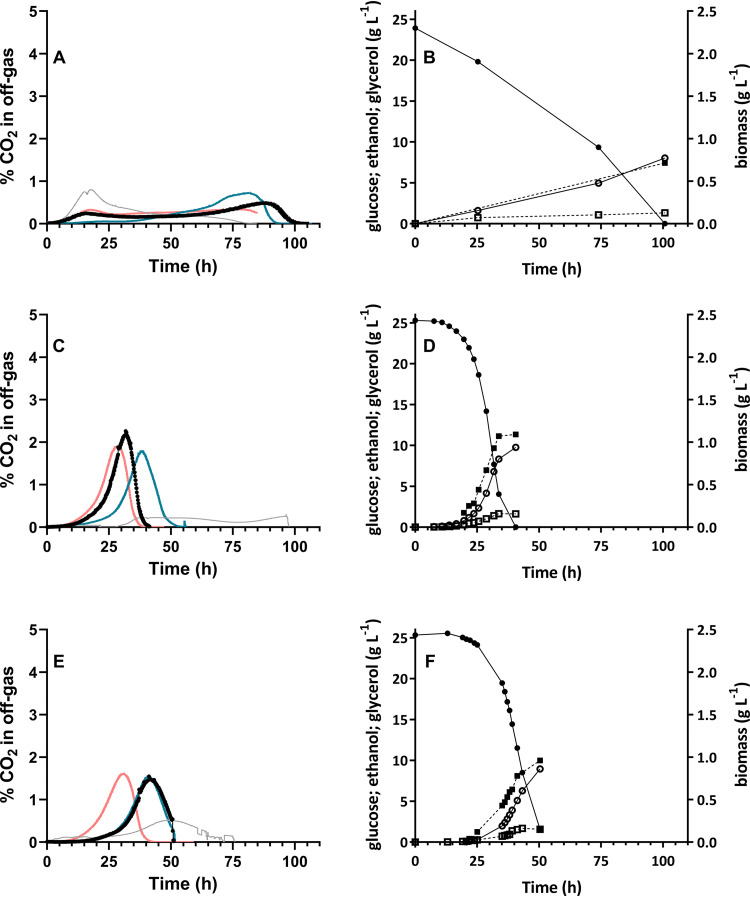
Anaerobic sequential batch bioreactor (SBR) cultivation of S. cerevisiae strains IMX585, IMX1438, and IMK870 without sterol supplementation. All panels represent data from a single representative SBR experiment performed at 30°C on SMD-urea without pH control. Data from replicate experiments are shown in Fig. S5. (A and B) Reference strain IMX585. (C and D) Strain IMX1438 (*sga1*Δ::*TtTHC1*). (E and F) Strain IMK870 (*sga1*Δ::*TtTHC1 erg1*Δ). (A, C, and E) Percentages of CO_2_ in the off-gas during the initial batch-cultivation cycle on medium without anaerobic growth factors (gray line) and the first (blue line), second (black line and dots), and third (red line) subsequent SBR cycles on medium supplemented only with Tween 80. (B, D, and F) Concentrations of glucose (closed circles), biomass (closed squares), ethanol (open circles), and glycerol (open squares).

CO_2_ off-gas profiles confirmed the inability of S. cerevisiae reference strain IMX585 to grow exponentially on SMD-urea without ergosterol (Fig. 4A; see also Fig. S5). Slow consumption of glucose by this strain took approximately 100 h (Fig. 4B). Based on the initial and final concentrations of glucose and biomass in these experiments (Table S1), the estimated specific growth rate and biomass yield over this period were 0.034 ± 0.000 h^−1^ and 0.027 ± 0.002 g biomass (g glucose)^−1^, respectively (Table 1). These values were 87% and 69% lower, respectively, than those of SBR cultures of this reference strain supplemented with both Tween 80 and ergosterol (Table 1). Similar residual growth rates of S. cerevisiae strains in anaerobic bioreactor cultures on synthetic medium without anaerobic growth factors were previously attributed to low levels of contamination with oxygen (46, 47).

In contrast to the IMX585 reference strain, strain IMX1438 (*sga1*Δ::*TtTHC1*) showed exponential anaerobic growth in the absence of sterol supplementation, at a specific growth rate of 0.15 h^−1^ (Fig. 4C) (Table 1; see also Fig. S5). Although its specific growth rate and biomass yield under these conditions were 38% and 37% lower, respectively, than those of corresponding ergosterol-supplemented SBR cultures (Table 1), glucose was completely consumed within 45 h (Fig. 4D). Consistent with the observed lower biomass yield on glucose, the ethanol yield of the SBR cultures grown without sterol supplementation was 5.4% higher than that of sterol-supplemented cultures (Table 1).

Squalene contents of biomass from anaerobic SBR cultures of the IMX585 reference strain were 3.8-fold lower in ergosterol-supplemented cultures than in cultures grown on sterol-free SMD-urea, while lanosterol contents were not significantly different (Fig. 2A; see also Table S2). This observation is consistent with a previously reported mechanism for ergosterol-induced degradation of β-hydroxy β-methylglutaryl-CoA (HMG-CoA) reductase, leading to lower squalene levels in sterol-supplemented cultures (48, 49). Anaerobic SBR cultures of strain IMX1438 (*sga1*Δ::*TtTHC1*) grown on sterol-free SMD-urea showed 2.4-fold-higher tetrahymanol levels (1.13 ± 0.05 mg [g biomass]^−1^) than corresponding sterol-supplemented cultures. The squalene levels in these cultures were not substantially different. However, the level of lanosterol was 2.4-fold higher in the cultures grown in the absence of a sterol source. No clear differences in fatty acid chain length or degree of desaturation were observed between strains or sterol-supplementation regimes. However, the IMX585 reference strain showed 20% lower total fatty acid content in the slow-growing sterol-free cultures than in sterol-supplemented cultures. In contrast, strain IMX1438 (*sga1*Δ::*TtTHC1*) showed 40% higher fatty acid content when grown in sterol-free medium (Fig. 2B; see also Table S3).

### Sterol-independent anaerobic growth of a tetrahymanol-expressing strain lacking a functional sterol-biosynthesis pathway.

Even when extensive measures are implemented to achieve anaerobiosis, it is notoriously difficult to fully eliminate oxygen entry into laboratory bioreactors (46, 47, 50, 51). Indeed, low levels of residual synthesis of unsaturated fatty acids were observed in the anaerobic SBR setups used in the present study in the absence of Tween 80 (46). No ergosterol was detected in biomass of strain IMX1438 (*sga1*Δ::*TtTHC1*) or the IMX585 reference strain, taken from anaerobic SBR cultures grown on SMD-urea without ergosterol (Fig. 2A; see also Table S2). However, detection of small amounts of lanosterol, the first cyclic intermediate in the ergosterol biosynthesis pathway (Fig. 1), indicated a minor leakage of oxygen into the cultures.

The squalene epoxidase Erg1 catalyzes the first step in sterol synthesis from squalene (Fig. 1). To eliminate any residual formation of sterols caused by oxygen entry into the bioreactors, strain IMK870 (*sga1*Δ::*TtTHC1 erg1*Δ) was constructed. Despite the absence of a functional sterol synthesis pathway, strain IMK870 grew exponentially in anaerobic SBR cultures on SMD-urea without ergosterol (Fig. 4E; see also Fig. S5) and the glucose was completely consumed within 55 h (Fig. 4F). The specific growth rate of strain IMK870 in these cultures was 27% lower than that of strain IMX1438 (*sga1*Δ::*TtTHC1 ERG1*), while the biomass and ethanol yields of the two strains were not significantly different (Table 1). Neither ergosterol nor lanosterol was detected in anaerobically grown biomass of strain IMK870 (Fig. 2A; see also Fig. S2 and Table S2), while the squalene levels were approximately 2.7-fold higher in strain IMK870 than in strain IMX1438 (*sga1*Δ::*TtTHC1 ERG1*) (Fig. 2A) in the absence of sterol supplementation. Fatty acid contents and compositions of anaerobically grown biomass of the two strains did not show marked differences (Fig. 2B; see also Table S3).

### Aerobic, sterol-free growth of an *erg1* deletion mutant expressing *TtTHC1*.

To investigate whether tetrahymanol production would allow aerobic, sterol-independent growth of strain IMK870 (*sga1*Δ::*TtTHC1 erg1*Δ), an anaerobic preculture on sterol-free medium was used to inoculate parallel aerobic cultures on SMD-urea. To investigate the ability of strain IMK870 to grow on nonfermentable carbon sources, additional experiments were performed on synthetic medium supplemented with a mixture of ethanol and glycerol (SMEG-urea). Strains IMX585 and IMX1438 (*sga1*Δ::*TtTHC1*) were included as references.

On SMD-urea, strains IMX585 and IMX1438 rapidly initiated exponential growth (Table 2; see also Fig. S3). Strain IMK870 (*sga1*Δ::*TtTHC1 erg1*Δ) showed a lag phase of approximately 20 h, after which its specific growth rate was 0.14 ± 0.00 h^−1^. For over 150 h, strain IMK870 did not show detectable growth on SMEG-urea, suggesting a loss of respiratory capacity. In contrast, both reference strains started growing on these nonfermentable carbon sources after a short lag phase.

**TABLE 2:** Specific growth rates of S. cerevisiae strains in aerobic batch cultures^*a*^

Strain	Specific growth rate (h^−1^)	
	Glucose	Ethanol/glycerol
IMX585	0.33 ± 0.01	0.19 ± 0.00
IMX1438 (*sga1*Δ::*TtTHC1*)	0.35 ± 0.01	0.17 ± 0.01
IMK870 (*sga1*Δ::*TtTHC1 erg1*Δ)	0.14 ± 0.00	No growth

a Strains were grown aerobically in a Growth Profiler 960 in 96-well plates on synthetic medium with urea as the nitrogen source, and with either glucose or a mixture of ethanol and glycerol as the carbon source, at 30°C. Specific growth rates represent averages ± standard errors of the means of measurements of results from 6 individual wells for each combination of medium composition and yeast strain. Since the biomass concentrations were estimated by image analysis (see Materials and Methods), estimated specific growth rates may not precisely match those that were measured in shake-flask cultures.

**FIG 5:**
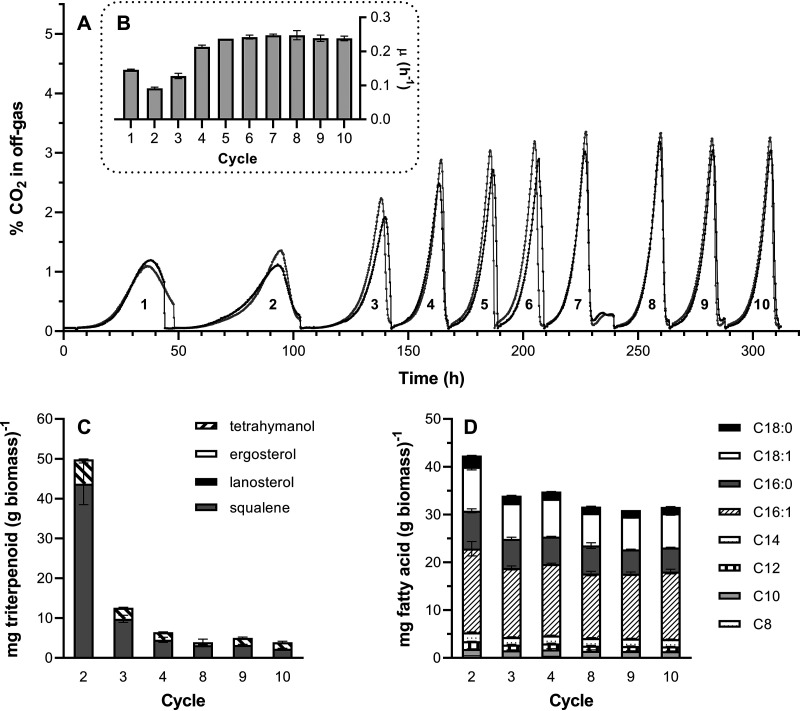
Aerobic sequential batch reactor experiments performed with S. cerevisiae IMK870 and analysis of triterpenoid fraction and total fatty acid composition of biomass by GC-FID. All panels represent data from two replicate aerobic bioreactor experiments performed at 30°C on SMD with ammonium as the nitrogen source and pH control at 5.0. (A) Percentage of CO_2_ in the off-gas during 10 subsequent batch cycles of two replicate experiments. (B) Specific growth rates estimated from CO_2_ production during 10 subsequent batch cycles. (C) Composition of the triterpenoid fraction of biomass harvested at the end of the indicated batch cycle. (D) Total fatty acid composition of biomass harvested at the end of the indicated batch cycle.

To explore whether aerobic growth of strain IMK870 (*sga1*Δ::*TtTHC1 erg1*Δ) was caused by carryover of small amounts of sterols from the preculture, its aerobic growth was further studied in aerobic SBR experiments. Over 10 consecutive SBR cycles, CO_2_ profiles indicated an increase of the specific growth rate in sterol-free medium from 0.14 h^−1^ in the first batch to 0.25 h^−1^ in the 10th cycle (Fig. 5A and B). Analysis of the triterpenoid fraction of aerobically grown biomass, performed in cycles 2 to 4 and 8 to 10, did not reveal the presence of sterols (Fig. 5C). Over the course of the first 4 SBR cycles, squalene content decreased from approximately 45 mg (g biomass)^−1^ to below 10 mg (g biomass)^−1^. The amount of tetrahymanol also decreased slightly but did so to a lesser extent. The fatty acid levels and compositions of the aerobic SBR cultures remained nearly unchanged through the course of the aerobic SBR experiments (Fig. 5D).

## DISCUSSION

This study demonstrated that expression of a heterologous squalene-tetrahymanol cyclase (STC) in S. cerevisiae enabled production of the pentacyclic triterpenoid tetrahymanol and allowed sterol-independent fermentative growth of this yeast under aerobic and anaerobic conditions.

The ability of eukaryotes to grow in the absence of sterol synthesis or supplementation is rare, with Neocallimastigomycota as a prominent exception. Horizontal gene transfer of a prokaryotic STC gene into these deep-branching anaerobes has been interpreted as a key evolutionary adaptation to life in the essentially anaerobic environment of the gut of large herbivores (32, 38). Due to the lack of efficient genetic tools (31), the physiological relevance of STC has not yet been experimentally verified in Neocallimastigomycota. By simulating acquisition of an STC gene through a horizontal gene transfer event in a yeast model, this study experimentally demonstrated that acquisition of a functional STC gene by a fermentative eukaryote confers an immediate advantage in anaerobic environments in which sterols are either absent or growth limiting.

*In vitro* studies demonstrated previously that sterol insertion into phospholipid membranes leads to denser membrane packing and reduced solute permeability (2, 52, 53). Increased permeability of sterol-depleted, tetrahymanol-containing yeast membranes to protons and/or other solutes may therefore have contributed to the reduced biomass yields and growth rates of *TtTHC1*-expressing S. cerevisiae strains in sterol-free media (Table 1). In addition, suboptimal growth characteristics of sterol-free, tetrahymanol-producing yeast cultures may be related to a wide range of other cellular processes that were shown to be affected by sterol composition in experiments performed with yeast sterol-biosynthesis mutants and sterol supplementation (7). For example, the inability of S. cerevisiae IMK870 (*sga1*Δ::*TtTHC1 erg1*Δ) to grow on the nonfermentable carbon sources ethanol and glycerol is consistent with a reported increased loss of mitochondrial DNA and mitochondrial function in response to reduced ergosterol content (54).

In eukaryotes that acquired a prokaryotic STC gene by horizontal gene transfer, subsequent evolutionary adaptations may have compensated for physiological disadvantages of sterol replacement. *Tetrahymena* species, in which tetrahymanol can be readily replaced by exogenous ergosterol (55), modify their fatty acid composition in response to sterol availability. Tetrahymanol-containing membranes of these protists contain fatty acids with a shorter acyl chain length and a lower degree of unsaturation than ergosterol-containing membranes, with a preference for the Δ^6,9^ isoform of C_18:2_ over the Δ^9,12^ isoform (56). While the total fatty acid content of *TtTHC1*-expressing S. cerevisiae was higher during anaerobic growth in sterol-free medium than in sterol-supplemented cultures (Fig. 2B; see also Table S3 in the supplemental material), no clear differences in fatty acid composition were observed. However, it should be noted that Tween 80, the source of unsaturated fatty acids in the anaerobic cultures, mainly provides oleic acid (C_18:1_). Sterol-deficient, tetrahymanol-producing S. cerevisiae strains provide an interesting platform for further studies on the combined impacts of triterpenoid and lipid compositions of fungal membranes on cellular robustness.

Despite extensive measures to prevent oxygen entry, small amounts of lanosterol, whose synthesis from squalene requires oxygen (Fig. 1), were detected in anaerobic SBR cultures of S. cerevisiae strains with an intact sterol synthesis pathway (Fig. 2; see also Fig. S2 and Table S2 in the supplemental material). This observation was in line with literature citing the technical challenges of anaerobic bioreactor cultivation of S. cerevisiae (46, 47, 50) and left the possibility that trace amounts of lanosterol and other sterols, whose levels were below the detection threshold of sterol analysis by gas chromatography (GC), might still be synthesized and contribute to the observed growth. Experiments performed with anaerobic and aerobic sequential batch reactors (SBRs) of a *TtTHC1*-expressing strain in which the *ERG1* gene had been deleted ruled out this possibility. In addition, based on an initial ergosterol level of 1.5 mg (g biomass)^−1^ (Table S2), a total of 3.4 × 10^10^ cells (g biomass)^−1^ (57), and an average of 4.76 generations for each SBR cycle (Table S1), the number of molecules per cell was on the order of 10^3^ after three SBR cycles and less than one molecule of ergosterol per cell remained after five SBR cycles (see “Calculations S1” in the supplemental material). Our results therefore demonstrated fully sterol-independent growth of *TtTHC1*-expressing *erg1*Δ S. cerevisiae.

Early studies in which specific sterols were added to oxygen-deprived S. cerevisiae cultures indicated that the hydroxyl group at the C-3 position and the configuration at the C-24 position of sterols were crucial for supporting anaerobic growth (23, 58). However, a large fraction of the required sterols were able to be replaced by cholesterol or any of a variety of other sterols lacking these configurations, as long as small quantities of ergosterol were also added (59). Similar conclusions were drawn based on experiments performed with aerobic cultures of sterol-auxotrophic S. cerevisiae strains (24, 60). These requirements for small amounts of ergosterol or closely related sterols, estimated at 1 × 10^7^ to 2 × 10^7^ molecules per cell, were proposed to reflect specific sterol-protein interactions (59). Our results indicate either that production of tetrahymanol can circumvent these requirements or that, in the strain background and under the experimental conditions used in the present study, a strict requirement for specific sterols does not exist. An apparent discrepancy with a previous study (61), which reported that exogenous tetrahymanol did not support anaerobic growth of S. cerevisiae on sterol-free medium, is likely to reflect an inability of the S. cerevisiae Aus1 and Pdr11 sterol transporters (20, 62) to transport tetrahymanol.

The increase, occurring over only 10 cycles of aerobic SBR cultivation, in the specific growth rate of a *TtTHC1*-expressing *erg1*Δ strain (Fig. 5) reflects either a physiological adaptation or rapid laboratory evolution. The accompanying decrease of the cellular content of squalene might be related to recently reported negative impacts of squalene accumulation in the S. cerevisiae plasma membrane (63). Dedicated laboratory evolution experiments performed under different environmental stress conditions, followed by whole-genome sequencing (64, 65), offer interesting possibilities to explore the genetic requirements for fast, robust growth of sterol-independent strains. In combination with the rapidly increasing knowledge on genome sequences of Neocallimastigomycota, such experiments may further extend our understanding of how anaerobic lifestyles have evolved in naturally occurring anaerobic fungi. In addition, they will provide valuable information for the design and construction of robust, sterol-independent yeast strains for application in anaerobic industrial processes.

## MATERIALS AND METHODS

### Strains, maintenance, and media.

The Saccharomyces cerevisiae strains used and constructed in this study (Table 3) were derived from the CEN.PK lineage (66, 67). Stock cultures were propagated in synthetic media (SM) (68) or in complex media (YP; 10 g liter^−1^ Bacto yeast extract [BD Biosciences, Franklin Lakes, NJ], 20 g liter^−1^ Bacto peptone [BD Biosciences]). Both types of media were autoclaved at 121°C, after which they were supplemented with 20 g liter^−1^ glucose from a concentrated solution and separately autoclaved at 110°C, resulting in SMD and YPD, respectively. Shake-flask and bioreactor experiments were performed in synthetic medium or in synthetic urea medium (SMD-urea [69]) supplemented with 20 g liter^−1^ glucose or with a mixture of 8.76 g liter^−1^ ethanol and 8.76 g liter^−1^ glycerol as the carbon sources (SMEG-urea). Where indicated, media were supplemented with 10 mg liter^−1^ ergosterol (Sigma-Aldrich, St. Louis, MO) (≥95% pure) and/or 420 mg liter^−1^ Tween 80 (polyethylene glycol sorbate monooleate; Merck, Darmstadt, Germany). Concentrated stock solutions of these supplements contained 8.4 g of Tween 80 and/or 0.2 g of ergosterol added to 17 ml ethanol and were heated at 80°C for 20 min prior to addition to growth media. Gas chromatography analysis with flame-ionization detection (GC-FID) of this concentrated Tween 80 stock solution did not reveal any contamination with sterols (see Fig. S1 in the supplemental material). Stock cultures of Escherichia coli DH5α and derived strains were grown in lysogeny broth (LB, 10 g liter^−1^ Bacto tryptone, 5 g liter^−1^ Bacto yeast extract, 5 g liter^−1^ NaCl [J.T. Baker, Avantor, Radnor, PA]) supplemented with 100 mg liter^−1^ ampicillin. After addition of sterile glycerol (30% [vol/vol]), samples of S. cerevisiae and E. coli stock cultures were frozen and stored at –80°C.

**TABLE 3:** Saccharomyces cerevisiae strains used in this study

Strain	Relevant genotype	Parental strain	Reference or source
IMX585	*MAT*α *can1*Δ::*cas9-natNT2*	CEN.PK113-7D	45
IMX1438	*MAT*α *can1*Δ::*cas9-natNT2 sga1*Δ::*TtTHC1*	IMX585	This study
IMK870	*MAT*α *can1*Δ::*cas9-natNT2 sga1*Δ::*TtTHC1 erg1*Δ::*KanMX*	IMX1438	This study

### Molecular biology techniques.

DNA fragments used for construction of plasmids and expression cassettes were amplified with Phusion high-fidelity DNA polymerase (Thermo Scientific, Waltham, MA) according to the manufacturer’s protocol and with PAGE-purified oligonucleotide primers (Sigma-Aldrich, St. Louis, MO). Diagnostic PCR was performed with DreamTaq PCR master mix (Thermo Scientific) following the manufacturer’s protocol and with desalted oligonucleotide primers (Sigma-Aldrich). PCR-amplified linear integration cassettes were purified from 1% (wt/vol) agarose gels using a Zymoclean gel DNA recovery kit (Zymo Research, Irvine, CA, USA). E. coli DH5α was transformed by electroporation with a MicroPulser electroporator (Bio-Rad, Hercules, CA). Plasmids were isolated from overnight E. coli cultures on LB with ampicillin by the use of a GenElute plasmid miniprep kit (Thermo Scientific). Chemical transformation of S. cerevisiae was performed as described previously by Gietz and Woods (70).

### Plasmid construction.

Plasmids and oligonucleotide primers used and/or constructed in this study are indicated in Table 4 and Table 5, respectively. The coding sequence of Tetrahymena thermophila squalene-tetrahymanol cyclase gene *THC1* (GenBank accession no. XM_001026696.2) was subjected to codon optimization for expression in S. cerevisiae using the Jcat algorithm (71). The codon-optimized coding sequence, flanked by 20-bp sequences for PCR amplification, was synthesized by GeneArt (Regensburg, Germany) and delivered in the pMK-RQ vector. Flanking sequences with homology to *TEF1* promoter and *CYC1* terminator sequences were added by PCR performed with primer pair 10561/10543, using pUD696 as the template. p426-TEF was linearized by PCR amplification with primer pair 5921/10547, and the synthetic gene fragment was cloned between the *TEF1* promoter and *CYC1* terminator on this expression plasmid by the use of Gibson assembly master mix (New England Biolabs, Ipswich, MA) to yield pUDE666.

**TABLE 4:** Plasmids used in this study

Plasmid	Characteristics	Reference or source
pUD696	pMK-RQ GeneArt delivery vector with the squalene-tetrahymanol cyclase gene from T. thermophila and PCR flanking regions	GeneArt
p426-TEF	2μm ori, *URA3*, Sc*TEF1p*-mcs-*ScCYC1t*	78
pUDE666	2μm ori, *URA3*, Sc*TEF1p-TtTHC1-CYC1t*	This study
pUDR119	2μm ori, *amdSYM, SNR52p*-gRNA_SGA1_*-SUP4t*	79
pUG6	Amp^r^,^*a*^ Ag*TEF1p*-KanMX-*AgTEF1t*	74

a Amp^r^, ampicillin resistance.

**TABLE 5:** Oligonucleotide primers used in this study

Oligonucleotide	Sequence
3811	CTCGGTGAGTTTTCTCCTTCAT
3812	TAGATTGTCGCACCTGATTG
5921	AAAACTTAGATTAGATTGCTATGCTTTCTTTCTAATGAGC
7298	TTGTTCAATGGATGCGGTTC
7479	GGACGTTCCGACATAGTATC
9626	TTTACAATATAGTGATAATCGTGGACTAGAGCAAGATTTCAAATAAGTAACAGCAGCAAAGCTCATAGCTTCAAAATGTTTCTAC
10148	CTGCAAACGTGGTTGGGCTGGACGTTCCGACATAGTATCTAATCAATTTATAATATCAGACAAATTAAAGCCTTCGAGCG
10543	GCGTGAATGTAAGCGTGACATAACTAATTACATGATATCGACAAAGGAAAAGGGGCCTGTCGCGCAGATTAGCGAAGC
10547	TCATGTAATTAGTTATGTCACGC
10561	TTTTTTTACTTCTTGCTCATTAGAAAGAAAGCATAGCAATCTAATCTAAGTTTTAATTACGCGATACCCTGCGATCTTC
11371	TTACCCAGCTTTCGACAAGG
11372	ACCACCTTGAGCAACGATCC
11783	GCATGCCGTGGCTGCTCTCGGTCGGGTATAAGTCTTAGACAATAGTCTTACCTCGCATGTCGACATGGAGGCCCAGAATACC
11784	GGAAGTAATATCGTTAATTGATAACCGAATATGAATCTCAATGCATATTTTGAAGCATATCGAATCGACAGCAGTATAGC
12183	GTGGTTCAGGGCACTCTACG
12184	CGTTATCACCGTTCCTTTCC

### Strain construction.

S. cerevisiae IMX1438 (*sga1*Δ::*TtTHC1*) was constructed by Cas9-mediated genome editing (45). The expression cassette for integration of *TtTHC1* was amplified from pUDE666 using primer pair 9626/10148. Cotransformation of S. cerevisiae IMX585 with 400 ng of the expression cassette and 500 ng of pUDR119, followed by curing of pUDR119 with fluoroacetamide (72), yielded strain IMX1438. Correct integration of linear fragments was checked by colony PCR (73) using primer pairs 7298/7479, 7298/11372, and 7479/11371, binding in the regions flanking the integration locus and inside the linear integration fragment. S. cerevisiae IMK870 was constructed by deleting *ERG1* in strain IMX1438. A KanMX expression cassette conferring resistance to G418 (74) was amplified from pUG6 using primer pair 11783/11784. Strain IMX1438 was transformed with 1 μg of this fragment, followed by overnight recovery in YPD. Subsequent overnight anaerobic incubation in 20 ml of YPD with Tween 80, ergosterol, and 200 mg liter^−1^ G418 (Invivogen, Toulouse, France), incubated in an anaerobic chamber for 2 days prior to inoculation, was used to preselect correct mutants. Cells from these cultures were plated on YPD-Tween 80/ergosterol agar with 200 mg liter^−1^ G418 and placed in an anaerobic jar (article no. SÜ380902; Schütt-biotec, Munich, Germany), together with an Anaerocult A catalyst package (VWR International BV, Amsterdam, The Netherlands) to remove traces of oxygen. Single cell lines were obtained by restreaking colonies three times on selective media. Correct integration of the KanMX marker in the *ERG1* locus was verified by colony PCR with primer pairs 12183/12184, 12183/3812, and 12184/3811, binding in the regions flanking the integration locus and inside the KanMX cassette.

### Shake-flask cultivation.

Aerobic shake-flask cultures were grown in 500-ml round-bottom shake flasks containing 100 ml of liquid media in an Innova shaker incubator (New Brunswick Scientific, Edison, NJ) set at 30°C and 200 rpm. Anaerobic shake-flask experiments were performed in a Shel Lab Bactron BAC X-2E anaerobic workstation (Sheldon Manufacturing Inc., Cornelius, OR). Anaerobic cultures were grown at 30°C in 50-ml or 100-ml round-bottom shake flasks containing 40 ml or 80 ml liquid medium, respectively, placed on an IKA KS 260 Basic orbital shaker platform (Dijkstra Vereenigde BV, Lelystad, The Netherlands) set at 200 rpm.

### Anaerobic bioreactor cultivation.

Anaerobic sequential batch reactor (SBR) experiments were performed as previously described (46) in 2-liter bioreactors (Applikon, Delft, the Netherlands) with a working volume of 1.2 liters at 30°C and an initial pH of 6.0. Cultures were stirred at 800 rpm, and to minimize oxygen contamination, no active pH control was used. Cultures were grown on SMD-urea to minimize changes in culture pH (69). The outlet gas of the bioreactors was cooled to 4°C in a condenser and dried with a model PD-50T-12MPP dryer (Permapure, Lakewood, NJ) prior to analysis performed with an NGA 2000 Rosemount gas analyzer (Emerson, St. Louis, MO). SBR experiments were initiated with a batch-cultivation cycle on medium without ergosterol and Tween 80 to deplete endogenous reserves of these growth factors. On-line measurements of CO_2_ concentrations in the outlet gas of reactors were used to monitor growth. When the proportion of CO_2_ in the off-gas decreased to a level below 0.1%, three consecutive SBR cycles performed on SMD-urea, containing either both supplements or only Tween 80, were initiated. Each subsequent SBR cycle was initiated when the CO_2_ concentration in the outlet gas decreased to a level below 0.05%, indicating depletion of the growth-limiting nutrient, by largely emptying the reactor and refilling with fresh medium, resulting in a 48-fold dilution of the original culture. Before refilling was performed, the medium inlet tube was flushed with nitrogen gas to avoid influx of contamination by any oxygen that might have permeated into the tube during the preceding growth phase. The 5-liter glass medium reservoir from which the cultures were refilled was kept anaerobic by continuous sparging with N5.5 grade N_2_ (Linde Gas Benelux, Schiedam, The Netherlands).

Precultures were prepared for anaerobic SBR experiments by inoculation of aerobic shake-flask cultures on SMD with frozen stock cultures of S. cerevisiae strain IMX585 or strain IMX1438 or with an anaerobic shake-flask culture of strain IMK870 (80 ml in a 100-ml flask, incubated in an anaerobic chamber) on SMD-urea with Tween 80 and ergosterol. After overnight cultivation at 30°C, a sample from these cultures was used to inoculate a second preculture on the same medium. After at least two biomass doublings, biomass was harvested by centrifugation at 3,000 × *g*, washed with sterile demineralized water, and used to inoculate anaerobic bioreactors at an initial optical density at 660 nm (OD_660_) of 0.2.

### Aerobic bioreactor cultivation.

Aerobic SBR experiments were performed as described for the anaerobic SBR experiments, with the following modifications: cultures were grown on SMD, culture pH was controlled at 5.0 by automated addition of 2 M KOH, aerobic conditions were maintained by sparging with air at 0.5 liters min^−1^, and the glass 20-liter medium reservoir was not sparged with nitrogen gas. SBR cycles were initiated either manually or automatically when the CO_2_ percentage in the off-gas had decreased below 10% of the maximum value that was measured during exponential phase.

### Growth studies in Growth Profiler.

Precultures for aerobic growth studies in a Growth Profiler 960 shaker (EnzyScreen BV, Heemstede, The Netherlands) were prepared as aerobic shake-flask cultures on YPD for strains IMX585 and IMX1438 and as anaerobic shake-flask cultures on 80 ml YPD supplemented with Tween 80 and ergosterol for strain IMK870. After overnight cultivation at 30°C, samples from these cultures were used to inoculate a second shake-flask preculture, which contained either 20 ml of SMD-urea for the aerobic precultures or 40 ml of SMD-urea supplemented only with Tween 80 for the anaerobic preculture. After overnight cultivation at 30°C, these cultures were washed twice with synthetic medium without a carbon or nitrogen source and were concentrated to an OD_660_ of 10. Aliquots (5 μl) of these suspensions were used to inoculate 96-well microtiter plates (EnzyScreen; type CR1496dl) with final working volumes of 250 μl and containing either SMD-urea or SMEG-urea at an initial OD_660_ of 0.2. Microtiter plates were closed with a sandwich cover (EnzyScreen; type CR1296). Growth experiments were performed at 30°C and at 250 rpm, and images of cultures were made at 30-min intervals. Corrected green values were obtained by the use of software supplied and installed by the manufacturer and were directly used for conversion to OD equivalents based on a 16-point calibration, leading to the following equation:

$$\text{OD equivalent}=0.242\times {\left[\text{GV}\left(t\right)-{\text{GV}}_{\text{med}}\right]}^{0.591}+8.6\times 10^{-5}\times {\left[\text{GV}\left(t\right)-{\text{GV}}_{\text{med}}\right]}^{2}{}^{.83}+5.27\times 10^{-9}\times {\left[\text{GV}\left(t\right)-{\text{GV}}_{\text{med}}\right]}^{4.85}$$

in which GV(*t*) is the corrected green value measured in a well at time point *t* and GV_med_ is a green value obtained through a measurement of the contents of a plate filled with medium performed before inoculation. Only OD equivalent values between 1.0 and 10 were used to estimate growth rates.

### Analytical methods.

Metabolite concentrations in culture supernatants were analyzed by high-performance liquid chromatography (HPLC) as described previously (75). HPLC measurements of ethanol concentrations were corrected for ethanol evaporation as described previously (76) using an evaporation coefficient of 0.0062. Biomass dry weight measurements and total fatty acid contents of freeze-dried biomass (as fatty acid methyl esters) were analyzed as described previously (46). Isolation of the triterpenoid fraction of biomass through saponification with NaOH and subsequent extraction with *tert*-butyl-methyl ether (*t*BME) was performed, essentially as described previously (77), with the following modifications. Biomass was harvested at the end of a cultivation cycle and/or during the mid-exponential phase by centrifugation of 50 ml of culture broth (5 min at 3,000 × *g*) and washed once with demineralized water. After the biomass pellets were lyophilized overnight (Alpha 1-4 LD Plus freeze dryer; Christ, Osterode am Harz, Germany), 10 to 30 mg of lyophilized material was weighed and placed into Pyrex borosilicate glass methylation tubes (article no. 10044604; Thermo Fisher Scientific). Then, 1 ml of 2 M NaOH (article no. 72068; Sigma-Aldrich) was added and suspensions were heated for 1 h at 70°C. During incubation, cell suspensions were not sonicated but were subjected to vortex mixing for 20 s at 15-min intervals. After cooling to room temperature, the content of the tube was transferred to a 2-ml plastic tube (Greiner Bio-One, Alphen aan den Rijn, The Netherlands) containing 650 μl of *t*BME. Subsequent extraction with *t*BME was done according to a previously published protocol (77). After extraction, the dried sterol fraction was dissolved in a volume of 100 μl to 1 ml of *t*BME, to obtain a final lipid concentration within the range of 10 to 500 μg ml^−1^, and directly used for analysis, without trimethyl silylation. Sterols were analyzed by gas chromatography with flame ionization detection (GC-FID) on an Agilent Technologies 7890A GC-FID system equipped with an FID-1000-220 gas station (Parker Balston, Haverhill, MA, USA) and an Agilent Technologies 7693 autosampler. A VF-5ms column (Agilent part no. CP9013) (30 m, 0.25-mm internal diameter, 0.25-μm film thickness) was used, with N_2_ used as the carrier gas at a constant flow of 1 ml min^−1^. The initial oven temperature of 80°C was kept constant for 1 min after sample injection and was then increased to 280°C at 50°C min^−1^ and was finally increased to 320°C at 6°C min^−1^ and kept at 320°C for a further 15 min. The inlet temperature was set at 150°C and the FID temperature at 330°C. The GC-FID system was calibrated with standards of squalene (Sigma-Aldrich) (≥98%), ergosterol (Boom B.V.) (≥98%), cholesterol (Sigma-Aldrich) (≥99%), lanosterol (Sigma-Aldrich) (≥93%), 5α-cholestane (internal standard) (Sigma-Aldrich) (≥97%), and tetrahymanol (ALB Technologies) (≥99%), using a 10-point calibration curve for all compounds except lanosterol and 5α-cholestane (6-point and 5-point calibration curves, respectively). Data were adjusted for internal standard concentrations and are expressed as milligrams of sterol per gram of lyophilized biomass. The statistical significance of differences between data from sets of replicate experiments was assessed with unpaired two-tailed Student’s *t* tests and a threshold (*P*) value of 0.05.

## Supplementary Material

Supplemental file 1

## References

[1] Mannock DA, Lewis R, McMullen TPW, McElhaney RN 2010 The effect of variations in phospholipid and sterol structure on the nature of lipid-sterol interactions in lipid bilayer model membranes. Chem Phys Lipids 163:403–448. doi:10.1016/j.chemphyslip.2010.03.011. 20371224

[2] Bui TT, Suga K, Umakoshi H 2016 Roles of sterol derivatives in regulating the properties of phospholipid bilayer systems. Langmuir 32:6176–6184. doi:10.1021/acs.langmuir.5b04343. 27158923

[3] Simons K, Sampaio JL 2011 Membrane organization and lipid rafts. Cold Spring Harb Perspect Biol 3:a004697. doi:10.1101/cshperspect.a004697. 21628426 PMC3179338

[4] Mollinedo F 2012 Lipid raft involvement in yeast cell growth and death. Front Oncol 2:140. doi:10.3389/fonc.2012.00140. 23087902 PMC3467458

[5] Summons RE, Bradley AS, Jahnke LL, Waldbauer JR 2006 Steroids, triterpenoids and molecular oxygen. Philos Trans R Soc Lond B Biol Sci 361:951–968. doi:10.1098/rstb.2006.1837. 16754609 PMC1578733

[6] Parks LW, Smith SJ, Crowley JH 1995 Biochemical and physiological effects of sterol alterations in yeast - a review. Lipids 30:227–230. doi:10.1007/BF02537825. 7791530

[7] Johnston EJ, Moses T, Rosser SJ 2020 The wide-ranging phenotypes of ergosterol biosynthesis mutants, and implications for microbial cell factories. Yeast 37:27–44. doi:10.1002/yea.3452. 31800968

[8] Munn AL, Heese-Peck A, Stevenson BJ, Pichler H, Riezman H 1999 Specific sterols required for the internalization step of endocytosis in yeast. Mol Biol Cell 10:3943–3957. doi:10.1091/mbc.10.11.3943. 10564282 PMC25690

[9] Proszynski TJ, Klemm RW, Gravert M, Hsu PP, Gloor Y, Wagner J, Kozak K, Grabner H, Walzer K, Bagnat M, Simons K, Walch-Solimena C 2005 A genome-wide visual screen reveals a role for sphingolipids and ergosterol in cell surface delivery in yeast. Proc Natl Acad Sci U S A 102:17981–17986. doi:10.1073/pnas.0509107102. 16330752 PMC1312417

[10] Umebayashi K, Nakano A 2003 Ergosterol is required for targeting of tryptophan permease to the yeast plasma membrane. J Cell Biol 161:1117–1131. doi:10.1083/jcb.200303088. 12810702 PMC2172991

[11] Caspeta L, Chen Y, Ghiaci P, Feizi A, Buskov S, Hallström BM, Petranovic D, Nielsen J 2014 Altered sterol composition renders yeast thermotolerant. Science 346:75–78. doi:10.1126/science.1258137. 25278608

[12] Endo A, Nakamura T, Shima J 2009 Involvement of ergosterol in tolerance to vanillin, a potential inhibitor of bioethanol fermentation, in *Saccharomyces cerevisiae*. FEMS Microbiol Lett 299:95–99. doi:10.1111/j.1574-6968.2009.01733.x. 19686341

[13] Liu G, Chen Y, Færgeman NJ, Nielsen J 2017 Elimination of the last reactions in ergosterol biosynthesis alters the resistance of *Saccharomyces cerevisiae* to multiple stresses. FEMS Yeast Res 17. doi:10.1093/femsyr/fox063. 28910986

[14] Abe F, Usui K, Hiraki T 2009 Fluconazole modulates membrane rigidity, heterogeneity, and water penetration into the plasma membrane in *Saccharomyces cerevisiae*. Biochemistry 48:8494–8504. doi:10.1021/bi900578y. 19670905

[15] Nowosielski M, Hoffmann M, Wyrwicz LS, Stepniak P, Plewczynski DM, Lazniewski M, Ginalski K, Rychlewski L 2011 Detailed mechanism of squalene epoxidase inhibition by terbinafine. J Chem Inf Model 51:455–462. doi:10.1021/ci100403b. 21229992

[16] Jia N, Arthington-Skaggs B, Lee W, Pierson CA, Lees ND, Eckstein J, Barbuch R, Bard M 2002 *Candida albicans* sterol C-14 reductase, encoded by the *ERG24* gene, as a potential antifungal target site. Antimicrob Agents Chemother 46:947–957. doi:10.1128/aac.46.4.947-957.2002. 11897574 PMC127109

[17] Vranová E, Coman D, Gruissem W 2013 Network analysis of the MVA and MEP pathways for isoprenoid synthesis. Annu Rev Plant Biol 64:665–700. doi:10.1146/annurev-arplant-050312-120116. 23451776

[18] Liu J-F, Xia J-J, Nie K-L, Wang F, Deng L 2019 Outline of the biosynthesis and regulation of ergosterol in yeast. World J Microbiol Biotechnol 35:98. doi:10.1007/s11274-019-2673-2. 31222401

[19] Andreasen AA, Stier T 1953 Anaerobic nutrition of *Saccharomyces cerevisiae* I ergosterol requirement for growth in a defined medium. J Cell Comp Physiol 41:23–36. doi:10.1002/jcp.1030410103. 13034889

[20] Wilcox LJ, Balderes DA, Wharton B, Tinkelenberg AH, Rao G, Sturley SL 2002 Transcriptional profiling identifies two members of the ATP-binding cassette transporter superfamily required for sterol uptake in yeast. J Biol Chem 277:32466–32472. doi:10.1074/jbc.M204707200. 12077145

[21] Lorenz RT, Parks LW 1991 Involvement of heme components in sterol metabolism of *Saccharomyces cerevisiae*. Lipids 26:598–603. doi:10.1007/BF02536423. 1779707

[22] Rodriguez RJ, Low C, Bottema CDK, Parks LW 1985 Multiple functions for sterols in *Saccharomyces cerevisiae*. Biochim Biophys Acta 837:336–343. doi:10.1016/0005-2760(85)90057-8. 3904834

[23] Pinto WJ, Nes WR 1983 Stereochemical specificity for sterols in *Saccharomyces cerevisiae*. J Biol Chem 258:4472–4476. 6339498

[24] Lorenz RT, Casey WM, Parks LW 1989 Structural discrimination in the sparking function of sterols in the yeast *Saccharomyces cerevisiae*. J Bacteriol 171:6169–6173. doi:10.1128/jb.171.11.6169-6173.1989. 2681155 PMC210486

[25] Stukey JE, Mcdonough VM, Martin CE 1989 Isolation and characterization of *OLE1*, a gene affecting fatty acid desaturation from *Saccharomyces cerevisiae*. J Biol Chem 264:16537–16544. 2674136

[26] Tehlivets O, Scheuringer K, Kohlwein SD 2007 Fatty acid synthesis and elongation in yeast. Biochim Biophys Acta 1771:255–270. doi:10.1016/j.bbalip.2006.07.004. 16950653

[27] Varela C, Torrea D, Schmidt SA, Ancin-Azpilicueta C, Henschke PA 2012 Effect of oxygen and lipid supplementation on the volatile composition of chemically defined medium and chardonnay wine fermented with *Saccharomyces cerevisiae*. Food Chem 135:2863–2871. doi:10.1016/j.foodchem.2012.06.127. 22980883

[28] Depraetere SA, Delvaux F, Schutter D, De Williams IS, Winderickx J, Delvaux FR 2008 The influence of wort aeration and yeast preoxygenation on beer staling processes. Food Chem 107:242–249. doi:10.1016/j.foodchem.2007.08.023.

[29] David MH, Kirsop BH 1973 Yeast growth in relation to the dissolved oxygen and sterol content of wort. J Inst Brew 79:20–25. doi:10.1002/j.2050-0416.1973.tb03491.x.

[30] Larue F, Lafon-Lafourcade S, Ribereau-Gayon P 1980 Relationship between the sterol content of yeast cells and their fermentation activity in grape must. Appl Environ Microbiol 39:808–811. doi:10.1128/AEM.39.4.808-811.1980. 16345545 PMC291423

[31] Hooker CA, Lee KZ, Solomon KV 2019 Leveraging anaerobic fungi for biotechnology. Curr Opin Biotechnol 59:103–110. doi:10.1016/j.copbio.2019.03.013. 31005803

[32] Murphy CL, Youssef NH, Hanafy RA, Couger MB, Stajich JE, Wang Y, Baker K, Dagar SS, Griffith GW, Farag IF, Callaghan TM, Elshahed MS 2019 Horizontal gene transfer as an indispensable driver for evolution of Neocallimastigomycota into a distinct gut-dwelling fungal lineage. Appl Environ Microbiol 85:e00988-19. doi:10.1128/AEM.00988-19. 31126947 PMC6643240

[33] Liggenstoffer AS, Youssef NH, Couger MB, Elshahed MS 2010 Phylogenetic diversity and community structure of anaerobic gut fungi (phylum Neocallimastigomycota) in ruminant and non-ruminant herbivores. ISME J 4:1225–1235. doi:10.1038/ismej.2010.49. 20410935

[34] Ourisson G, Rohmer M, Poralla K 1987 Prokaryotic hopanoids and other polyterpenoid sterol surrogates. Annu Rev Microbiol 41:301–333. doi:10.1146/annurev.mi.41.100187.001505. 3120639

[35] Sáenz JP, Grosser D, Bradley AS, Lagny TJ, Lavrynenko O, Broda M, Simons K 2015 Hopanoids as functional analogues of cholesterol in bacterial membranes. Proc Natl Acad Sci U S A 112:11971–11976. doi:10.1073/pnas.1515607112. 26351677 PMC4586864

[36] Nes WD, Heftmann E 1981 A comparison of triterpenoids with steroids as membrane components. J Nat Prod 44:377–400. doi:10.1021/np50016a001.

[37] Youssef NH, Couger MB, Struchtemeyer CG, Liggenstoffer AS, Prade RA, Najar FZ, Atiyeh HK, Wilkins MR, Elshahed MS 2013 The genome of the anaerobic fungus *Orpinomyces* sp. strain C1A reveals the unique evolutionary history of a remarkable plant biomass degrader. Appl Environ Microbiol 79:4620–4634. doi:10.1128/AEM.00821-13. 23709508 PMC3719515

[38] Gruninger RJ, Puniya AK, Callaghan TM, Edwards JE, Youssef N, Dagar SS, Fliegerova K, Griffith GW, Forster R, Tsang A, McAllister T, Elshahed MS 2014 Anaerobic fungi (phylum Neocallimastigomycota): advances in understanding their taxonomy, life cycle, ecology, role and biotechnological potential. FEMS Microbiol Ecol 90:1–17. doi:10.1111/1574-6941.12383. 25046344

[39] Abe I 2007 Enzymatic synthesis of cyclic triterpenes. Nat Prod Rep 24:1311–1331. doi:10.1039/b616857b. 18033581

[40] Mallory FB, Gordon JT, Conner RL 1963 The isolation of a pentacyclic triterpenoid alcohol from a protozoan. J Am Chem Soc 85:1362–1363. doi:10.1021/ja00892a042.

[41] Zander JM, Caspi E, Pandey GN, Mitra CR 1969 The presence of tetrahymanol in *Oleandra wallichii*. Phytochemistry 8:2265–2267. doi:10.1016/S0031-9422(00)88195-9.

[42] Kleemann G, Kellner R, Poralla K 1994 Purification and properties of the squalene-hopene cyclase from *Rhodopseudomonas palustris*, a purple non-sulfur bacterium producing hopanoids and tetrahymanol. Biochim Biophys Acta 1210:317–320. doi:10.1016/0005-2760(94)90235-6. 8305486

[43] Bravo JM, Perzl M, Härtner T, Kannenberg EL, Rohmer M 2001 Novel methylated triterpenoids of the gammacerane series from the nitrogen-fixing bacterium *Bradyrhizobium japonicum* USDA 110. Eur J Biochem 268:1323–1331. doi:10.1046/j.1432-1327.2001.01998.x. 11231284

[44] Banta AB, Wei JH, Welander PV 2015 A distinct pathway for tetrahymanol synthesis in bacteria. Proc Natl Acad Sci U S A 112:13478–13483. doi:10.1073/pnas.1511482112. 26483502 PMC4640766

[45] Mans R, van Rossum HM, Wijsman M, Backx A, Kuijpers NGA, van den Broek M, Daran-Lapujade P, Pronk JT, van Maris AJA, Daran J-M 2015 CRISPR/Cas9: a molecular Swiss Army knife for simultaneous introduction of multiple genetic modifications in *Saccharomyces cerevisiae*. FEMS Yeast Res 15:fov004. doi:10.1093/femsyr/fov004. 25743786 PMC4399441

[46] Dekker WJC, Wiersma SJ, Bouwknegt J, Mooiman C, Pronk JT 2019 Anaerobic growth of *Saccharomyces cerevisiae* CEN.PK113-7D does not depend on synthesis or supplementation of unsaturated fatty acids. FEMS Yeast Res 19:foz060. doi:10.1093/femsyr/foz060. 31425603 PMC6750169

[47] da Costa BLV, Raghavendran V, Franco LFM, Chaves Filho A de B, Yoshinaga MY, Miyamoto S, Basso TO, Gombert AK 2019 Forever panting and forever growing: physiology of *Saccharomyces cerevisiae* at extremely low oxygen availability in the absence of ergosterol and unsaturated fatty acids. FEMS Yeast Res 19:foz054. doi:10.1093/femsyr/foz054. 31425576

[48] Hu Z, Bin He B, Long Ma B, Yunlong Sun B, Yali Niu B, Bin Zeng B 2017 Recent advances in ergosterol biosynthesis and regulation mechanisms in *Saccharomyces cerevisiae*. Indian J Microbiol 57:270–277. doi:10.1007/s12088-017-0657-1. 28904410 PMC5574775

[49] Pinto WJ, Lozano R, Nes WR 1985 Inhibition of sterol biosynthesis by ergosterol and cholesterol in *Saccharomyces cerevisiae*. Biochim Biophys Acta 836:89–95. doi:10.1016/0005-2760(85)90224-3. 3896318

[50] Visser W, Scheffers WA, Batenburg-Van Der Vegte WH, Van Dijken JP 1990 Oxygen requirements of yeasts. Appl Environ Microbiol 56:3785–3792. doi:10.1128/AEM.56.12.3785-3792.1990. 2082825 PMC185068

[51] da Costa BLV, Basso TO, Raghavendran V, Gombert AK 2018 Anaerobiosis revisited: growth of *Saccharomyces cerevisiae* under extremely low oxygen availability. Appl Microbiol Biotechnol 102:2101–2116. doi:10.1007/s00253-017-8732-4. 29397429

[52] Yeagle PL, Martin RB, Lala AK, Lin HK, Bloch K 1977 Differential effects of cholesterol and lanosterol on artificial membranes. Proc Natl Acad Sci U S A 74:4924–4926. doi:10.1073/pnas.74.11.4924. 270726 PMC432069

[53] Haines TH 2001 Do sterols reduce proton and sodium leaks through lipid bilayers? Prog Lipid Res 40:299–324. doi:10.1016/s0163-7827(01)00009-1. 11412894

[54] Cirigliano A, Macone A, Bianchi MM, Oliaro-Bosso S, Balliano G, Negri R, Rinaldi T 2019 Ergosterol reduction impairs mitochondrial DNA maintenance in *S. cerevisiae*. Biochim Biophys Acta Mol Cell Biol Lipids 1864:290–303. doi:10.1016/j.bbalip.2018.12.002. 30553056

[55] Conner RL, Mallory FB, Landrey JR, Ferguson KA, Kaneshiro ES, Ray E 1971 Ergosterol replacement of tetrahymanol in *Tetrahymena* membranes. Biochem Biophys Res Commun 44:995–1000. doi:10.1016/0006-291X(71)90810-2. 4108155

[56] Ferguson KA, Davis FM, Conner RL, Landrey JR, Mallory FB 1975 Effect of sterol replacement *in vivo* on the fatty acid composition of *Tetrahymena*. J Biol Chem 250:6998–7005. 808549

[57] Bisschops MMM, Zwartjens P, Keuter SGF, Pronk JT, Daran-Lapujade P 2014 To divide or not to divide: a key role of Rim15 in calorie-restricted yeast cultures. Biochim Biophys Acta 1843:1020–1030. doi:10.1016/j.bbamcr.2014.01.026. 24487068

[58] Nes WR, Sekula BC, Nes WD, Adler JH 1978 The functional importance of structural features of ergosterol in yeast. J Biol Chem 253:6218–6225. 355252

[59] Pinto WJ, Lozano R, Sekula BC, Nes WR 1983 Stereochemically distinct roles for sterol in *Saccharomyces cerevisiae*. Biochem Biophys Res Commun 112:47–54. doi:10.1016/0006-291x(83)91795-3. 6340686

[60] Rodriguez RJ, Taylor FR, Parks LW 1982 A requirement for ergosterol to permit growth of yeast sterol auxotrophs on cholestanol. Biochem Biophys Res Commun 106:435–441. doi:10.1016/0006-291x(82)91129-9. 6809003

[61] Nes WD, Janssen GG, Crumley FG, Kalinowska M, Akihisa T 1993 The structural requirements of sterols for membrane function in *Saccharomyces cerevisiae*. Arch Biochem Biophys 300:724–733. doi:10.1006/abbi.1993.1100. 8434952

[62] Li Y, Prinz WA 2004 ATP-binding cassette (ABC) transporters mediate nonvesicular, raft-modulated sterol movement from the plasma membrane to the endoplasmic reticulum. J Biol Chem 279:45226–45234. doi:10.1074/jbc.M407600200. 15316012

[63] Csáky Z, Garaiová M, Kodedová M, Valachovič M, Sychrová H, Hapala I 2020 Squalene lipotoxicity in a lipid droplet‐less yeast mutant is linked to plasma membrane dysfunction. Yeast 37:45–62. doi:10.1002/yea.3454. 31826302

[64] Mans R, Daran JMG, Pronk JT 2018 Under pressure: evolutionary engineering of yeast strains for improved performance in fuels and chemicals production. Curr Opin Biotechnol 50:47–56. doi:10.1016/j.copbio.2017.10.011. 29156423

[65] Sandberg TE, Salazar MJ, Weng LL, Palsson BO, Feist AM 2019 The emergence of adaptive laboratory evolution as an efficient tool for biological discovery and industrial biotechnology. Metab Eng 56:1–16. doi:10.1016/j.ymben.2019.08.004. 31401242 PMC6944292

[66] Entian KD, Kötter P 2007 25 Yeast genetic strain and plasmid collections. Methods Microbiol 36:629–666. doi:10.1016/S0580-9517(06)36025-4.

[67] Nijkamp JF, van den Broek M, Datema E, de Kok S, Bosman L, Luttik MA, Daran-Lapujade P, Vongsangnak W, Nielsen J, Heijne WH, Klaassen P, Paddon CJ, Platt D, Kötter P, van Ham RC, Reinders MJ, Pronk JT, de Ridder D, Daran J-M 2012 *De novo* sequencing, assembly and analysis of the genome of the laboratory strain *Saccharomyces cerevisiae* CEN.PK113-7D, a model for modern industrial biotechnology. Microb Cell Fact 11:36. doi:10.1186/1475-2859-11-36. 22448915 PMC3364882

[68] Verduyn C, Postma E, Scheffers WA, Van Dijken JP 1992 Effect of benzoic acid on metabolic fluxes in yeasts: a continuous-culture study on the regulation of respiration and alcoholic fermentation. Yeast 8:501–517. doi:10.1002/yea.320080703. 1523884

[69] Luttik MA, Kötter P, Salomons FA, van der Klei IJ, van Dijken JP, Pronk JT 2000 The *Saccharomyces cerevisiae ICL2* gene encodes a mitochondrial 2-methylisocitrate lyase involved in propionyl-coenzyme A metabolism. J Bacteriol 182:7007–7013. doi:10.1128/jb.182.24.7007-7013.2000. 11092862 PMC94827

[70] Gietz RD, Woods RA 2002 Transformation of yeast by lithium acetate/single-stranded carrier DNA/polyethylene glycol method. Methods Enzymol 313:87–96. doi:10.1016/s0076-6879(02)50957-5. 12073338

[71] Grote A, Hiller K, Scheer M, Münch R, Nörtemann B, Hempel DC, Jahn D 2005 JCat: a novel tool to adapt codon usage of a target gene to its potential expression host. Nucleic Acids Res 33:W526–W531. doi:10.1093/nar/gki376. 15980527 PMC1160137

[72] Solis-Escalante D, Kuijpers NGA, Bongaerts N, Bolat I, Bosman L, Pronk JT, Daran J-M, Daran-Lapujade P 2013 *amdSYM*, a new dominant recyclable marker cassette for *Saccharomyces cerevisiae*. FEMS Yeast Res 13:126–139. doi:10.1111/1567-1364.12024. 23253382 PMC3563226

[73] Lõoke M, Kristjuhan K, Kristjuhan A 2011 Extraction of genomic DNA from yeasts for PCR-based applications. Biotechniques 50:325–328. doi:10.2144/000113672. 21548894 PMC3182553

[74] Güldener U, Heck S, Fielder T, Beinhauer J, Hegemann JH 1996 A new efficient gene disruption cassette for repeated use in budding yeast. Nucleic Acids Res 24:2519–2524. doi:10.1093/nar/24.13.2519. 8692690 PMC145975

[75] Verhoeven MD, Lee M, Kamoen L, van den Broek M, Janssen DB, Daran J-M, van Maris AJA, Pronk JT 2017 Mutations in *PMR1* stimulate xylose isomerase activity and anaerobic growth on xylose of engineered *Saccharomyces cerevisiae* by influencing manganese homeostasis. Sci Rep 7:46155. doi:10.1038/srep46155. 28401919 PMC5388867

[76] Guadalupe-Medina V, Almering MJH, Van Maris AJA, Pronk JT 2010 Elimination of glycerol production in anaerobic cultures of a *Saccharomyces cerevisiae* strain engineered to use acetic acid as an electron acceptor. Appl Environ Microbiol 76:190–195. doi:10.1128/AEM.01772-09. 19915031 PMC2798634

[77] Müller C, Binder U, Bracher F, Giera M 2017 Antifungal drug testing by combining minimal inhibitory concentration testing with target identification by gas chromatography-mass spectrometry. Nat Protoc 12:947–963. doi:10.1038/nprot.2017.005. 28384139

[78] Mumberg D, Müller R, Funk M 1995 Yeast vectors for the controlled expression of heterologous proteins in different genetic backgrounds. Gene 156:119–122. doi:10.1016/0378-1119(95)00037-7. 7737504

[79] van Rossum HM, Kozak BU, Niemeijer MS, Duine HJ, Luttik MAH, Boer VM, Kötter P, Daran JMG, van Maris AJA, Pronk JT 2016 Alternative reactions at the interface of glycolysis and citric acid cycle in *Saccharomyces cerevisiae*. FEMS Yeast Res 16:fow017. doi:10.1093/femsyr/fow017. 26895788 PMC5815053

