# Supplementary material for: Squalene-Tetrahymanol Cyclase Expression Enables Sterol-Independent Growth of Saccharomyces cerevisiae
Source: Appl Environ Microbiol. 2020 Aug 18;86(17):e00672-20. doi: 10.1128/AEM.00672-20 (PMC7440791; doi:10.1128/AEM.00672-20)
Supplement: Supplemental file 1 [file AEM.00672-20-s0001.pdf]

Expression of a squalene-tetrahymanol cyclase enables sterol-independent growth of *Saccharomyces cerevisiae*

Sanne J. Wiersma, Christiaan Mooiman, Martin Giera, Jack T. Pronk

SUPPLEMENTARY MATERIAL

Table S1: Optical density measurements and metabolite consumption and production at start and end of anaerobic batch experiments. The measurements were done at the start and end of each batch cultivation. Data represent mean and standard error of the mean of three replicate experiments, unless otherwise indicated.

Experiment	Cycle	OD_{660}, start	OD_{660},end	Glucose, cons (mM)	Ethanol, prod (mM)	Glycerol, prod (mM)	Acetate, prod (mM)
IMX585, with sterols	CO	0.24 ± 0.01	5.64 ± 0.43	143.1 ± 1.3	162.0 ± 2.4	29.9 ± 0.3	9.9 ± 0.2
	1	0.16 ± 0.01	15.1 ± 0.25	131.2 ± 0.4	184.1 ± 1.2	17.1 ± 0.0	1.8 ± 0.1
	2	0.40 ± 0.05	13.9 ± 0.29	132.1 ± 1.1	180.7 ± 1.4	16.8 ± 0.1	2.0 ± 0.0
	3	0.35 ± 0.06	10.7 ± 1.5	130.7 ± 1.1	146.2 ± 1.8	16.9 ± 0.0	2.4 ± 0.2
IMX585, without sterols[a]	CO	0.21 ± 0.00	6.76 ± 0.83	136.9 ± 0.3	158.2 ± 2.8	28.7 ± 0.5	9.6 ± 0.3
	1	0.20 ± 0.01	6.14 ± 0.11	132.7 ± 0.3	180.5 ± 5.1	15.6 ± 0.0	2.8 ± 0.0
	2	0.16 ± 0.00	4.77 ± 0.37	133.0 ± 0.1	167.3 ± 7.2	14.8 ± 0.3	3.9 ± 0.4
	3[b]	*0.14 ± 0.02*	*3.47 ± 0.63*	*83.1 ± 18.0*	*109.3 ± 26.2*	*13.4 ± 0.5*	*3.6 ± 0.4*
IMX1438, with sterols	CO	0.24 ± 0.00	5.18 ± 0.6	115.5 ± 3.8	141.2 ± 4.1	20.6 ±0.5	2.8 ± 0.1
	1	0.16 ± 0.01	14.6 ± 0.3	132.6 ± 0.5	194.9 ± 0.9	18.0 ± 0.1	1.7 ± 0.1
	2	0.29 ± 0.04	15.4 ± 0.5	133.2 ± 0.5	189.9 ± 4.5	17.6 ± 0.2	1.5 ± 0.1
	3	0.32 ± 0.03	14.9 ± 0.0	131.9 ± 0.6	193.4 ± 1.7	16.2 ± 1.3	1.6 ± 0.0
IMX1438, without sterols	CO	0.13 ± 0.01	3.47 ± 0.08	78.9 ± 3.3	101.4 ± 2.4	15.9 ± 0.6	3.0 ± 0.1
	1	0.15 ± 0.30	6.85 ± 0.28	44.4 ± 1.0	211.8 ± 1.3	0.5 ± 0.1	1.0 ± 0.0
	2	0.21 ± 0.04	7.51 ± 0.25	139.5 ± 0.9	207.7 ± 3.4	18.5 ± 0.8	3.2 ± 0.2
	3	0.24 ± 0.04	6.71 ± 0.17	140.9 ± 0.2	210.9 ± 1.5	17.6 ± 0.2	3.7 ± 0.0
IMK870, without sterols[a]	CO	0.23 ± 0.02	5.65 ± 0.37	118.1 ± 4.9	163.1 ± 13.2	19.5 ± 0.3	2.0 ± 0.1
	1	0.18 ± 0.03	5.45 ± 0.30	135.3 ± 0.5	201.9 ± 0.6	17.1 ± 0.8	3.4 ± 0.1
	2	0.22 ± 0.02	5.66 ± 0.13	136.6 ± 4.6	188.3 ± 6.2	16.3 ± 0.8	3.9 ± 0.1
	3	0.21 ± 0.03	5.27 ± 0.41	142.3 ± 0.9	201.4 ± 11.2	20.2 ± 2.8	5.0 ± 0.7

[a] Two replicate experiments ; [b] Experiment was terminated untimely due to an electrical failure

Table S2: Composition of triterpenoid fraction of biomass during anaerobic SBR experiments. Unless otherwise indicated, data represent the average and standard error of the mean of three replicate experiments, in mg triterpenoid (g biomass)$^{-1}$.

Experiment	Squalene	Ergosterol	Lanosterol	Tetrahymanol
**IMX585**, With sterols	1.54 ± 0.30[b]	1.55 ± 0.25	0.28 ± 0.14	0
**IMX585**, Without sterols[a]	5.86 ± 0.63[b]	0	0.39 ± 0.39	0
**IMX1438**, With sterols[a]	1.14 ± 0.23	1.73 ± 0.34	0.32 ± 0.04[d]	0.47 ± 0.09[e]
**IMX1438**, Without sterols	0.84 ± 0.12[c]	0	0.76 ± 0.03[d]	1.13 ± 0.05[e]
**IMK870**, Without sterols[a]	2.29 ± 0.18[c]	0	0	1.48 ± 0.11

[a] Two replicate experiments

[b,c,d,e] Pairs of datasets of which the means were determined to be significantly different using a two-tailed unpaired Student's t-test with a p-value of 0.05

Table S3: Total fatty acid composition of biomass during anaerobic SBR experiments.

Data represent the mean and standard error of the mean of three replicate experiments, unless otherwise indicated, in mg fatty acid (g biomass)$^{-1}$.

**Experiment / Fatty acid**	**IMX585** With sterols	**IMX585**[a] Without sterols	**IMX1438** With sterols	**IMX1438** Without sterols	**IMK870**[a] Without sterols
**C8:0**	0.00 ±0.00	0.05 ± 0.02	0.00 ±0.00	0.04 ±0.02	0.01 ±0.00
**C10:0**	0.00 ±0.00	0.23 ± 0.00	0.00 ±0.00	0.76 ±0.06	0.52 ±0.05
**C12:0**	0.44 ±0.11	0.84 ± 0.02	0.35 ±0.01	1.08 ±0.06	1.09 ±0.03
**C14:0**	1.81 ±0.12	1.57 ± 0.02	1.93 ±0.11	2.92 ±0.10	2.76 ±0.05
**C16:1**	0.33 ±0.01	0.27 ± 0.01	0.36 ±0.00	0.15 ±0.02	0.19 ±0.04
**C16:0**	5.99 ±0.23	3.87 ± 0.16	6.83 ±0.29	8.37 ±0.25	8.24 ±0.06
**C18:1**	16.1 ±0.7	12.5 ± 0.1	14.4 ±0.5	19.9 ±0.7	20.52 ±0.59
**C18:0**	0.84 ±0.04	0.98 ± 0.09	0.95 ±0.03	1.33 ±0.07	1.34 ±0.08
**Total**	25.5 ±1.1[b]	20.4 ±0.3[b]	24.8 ±0.8[c]	34.6 ±1.2[c]	34.7 ±0.4

[a] Two replicate experiments

[b,c] Pairs of datasets of which the means were determined to be significantly different using a two-tailed unpaired Student's t-test with a p-value of 0.05

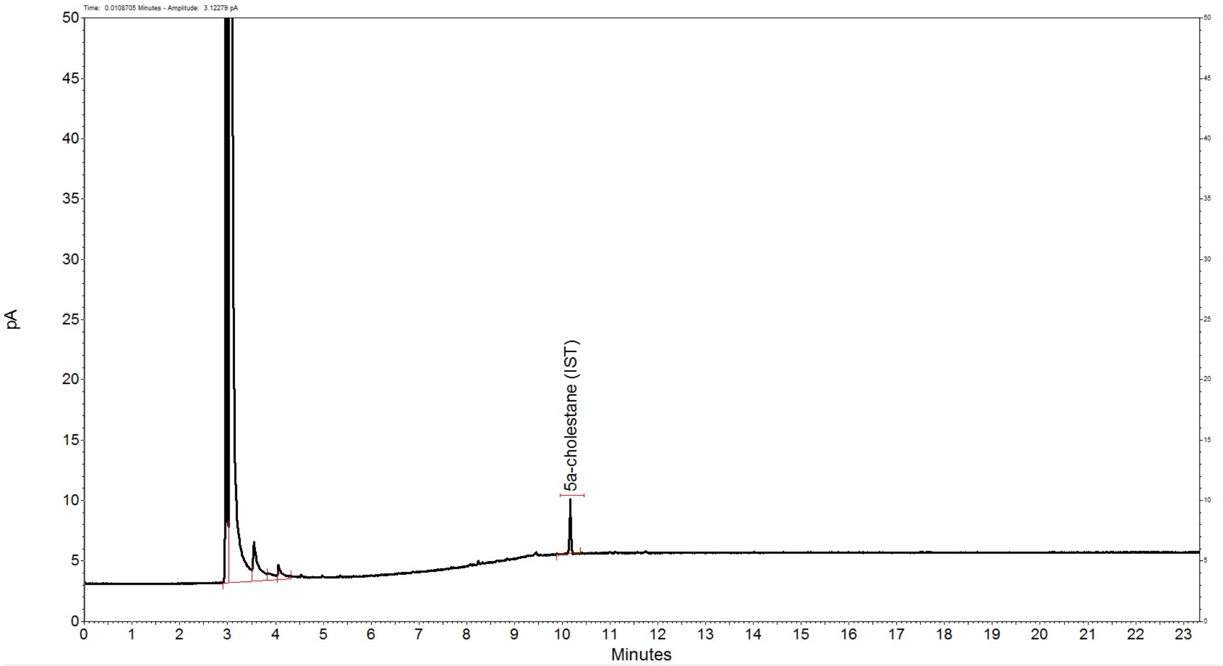

Figure S1. **Quality control of Tween 80.** 250 μL of a Tween 80 stock solution, containing 105 μg of Tween 80, was processed according to the protocol for isolation of the triterpenoid fraction of yeast biomass and analysed by GC-FID. 50 μg of 5α-cholestane was added as internal standard. Retention times of squalene, ergosterol, lanosterol and tetrahymanol are 9.49, 12.07, 12.81, 14.28 and 18.37 minutes, respectively.

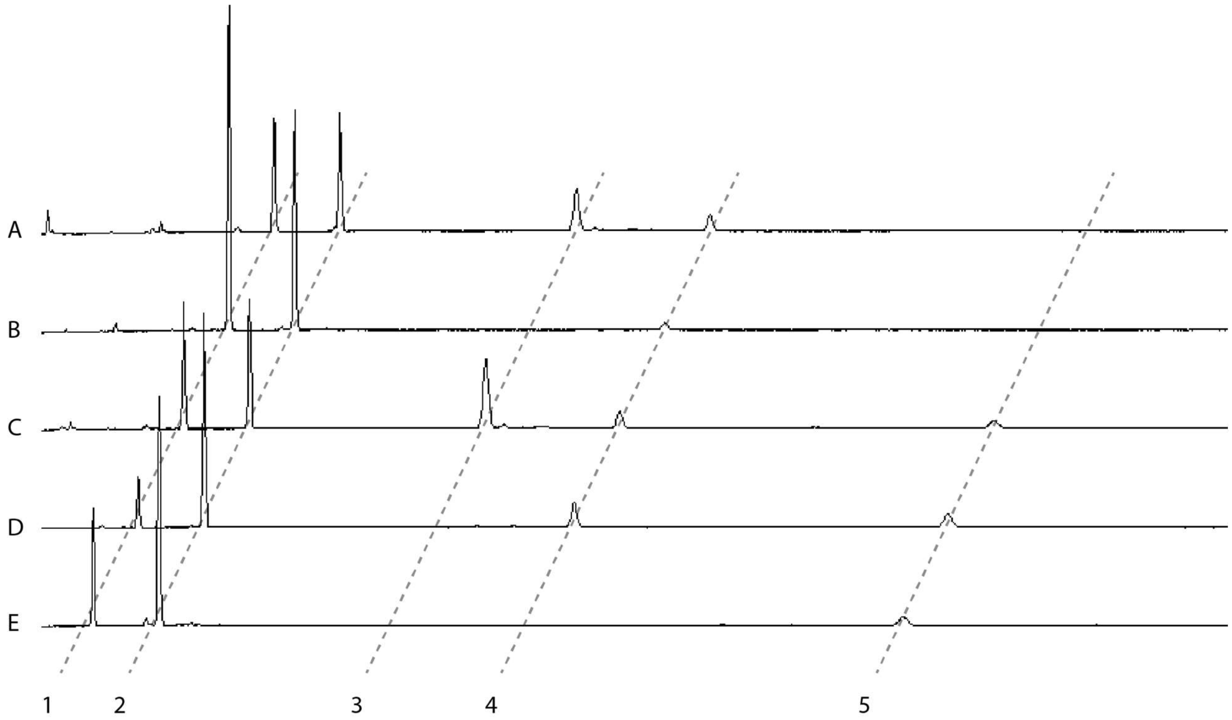

Figure S2: Gas chromatograms of analysis of triterpenoid fraction of biomass harvested from anaerobic batch cultures. Letters A-E indicate one representative chromatogram of three replicate experiments. **A:** IMX585 (reference strain) supplemented with ergosterol; **B:** IMX585 (reference strain) without ergosterol; **C:** IMX1438 (*sga1Δ::TtTHC1*) supplemented with ergosterol; **D:** IMX1438 (*sga1Δ::TtTHC1*) without ergosterol; **E:** IMK870 (*sga1Δ::TtTHC1 erg1Δ*) without ergosterol. Numbers 1-5 indicate identified compounds in the triterpenoid fraction; RRT = relative retention time. **1:** squalene (RRT 0.93); **2:** 5α-cholestane, internal standard (RRT 1.0); **3:** ergosterol (RRT 1.26); **4:** lanosterol (RRT 1.40); **5:** tetrahymanol (RRT 1.80).

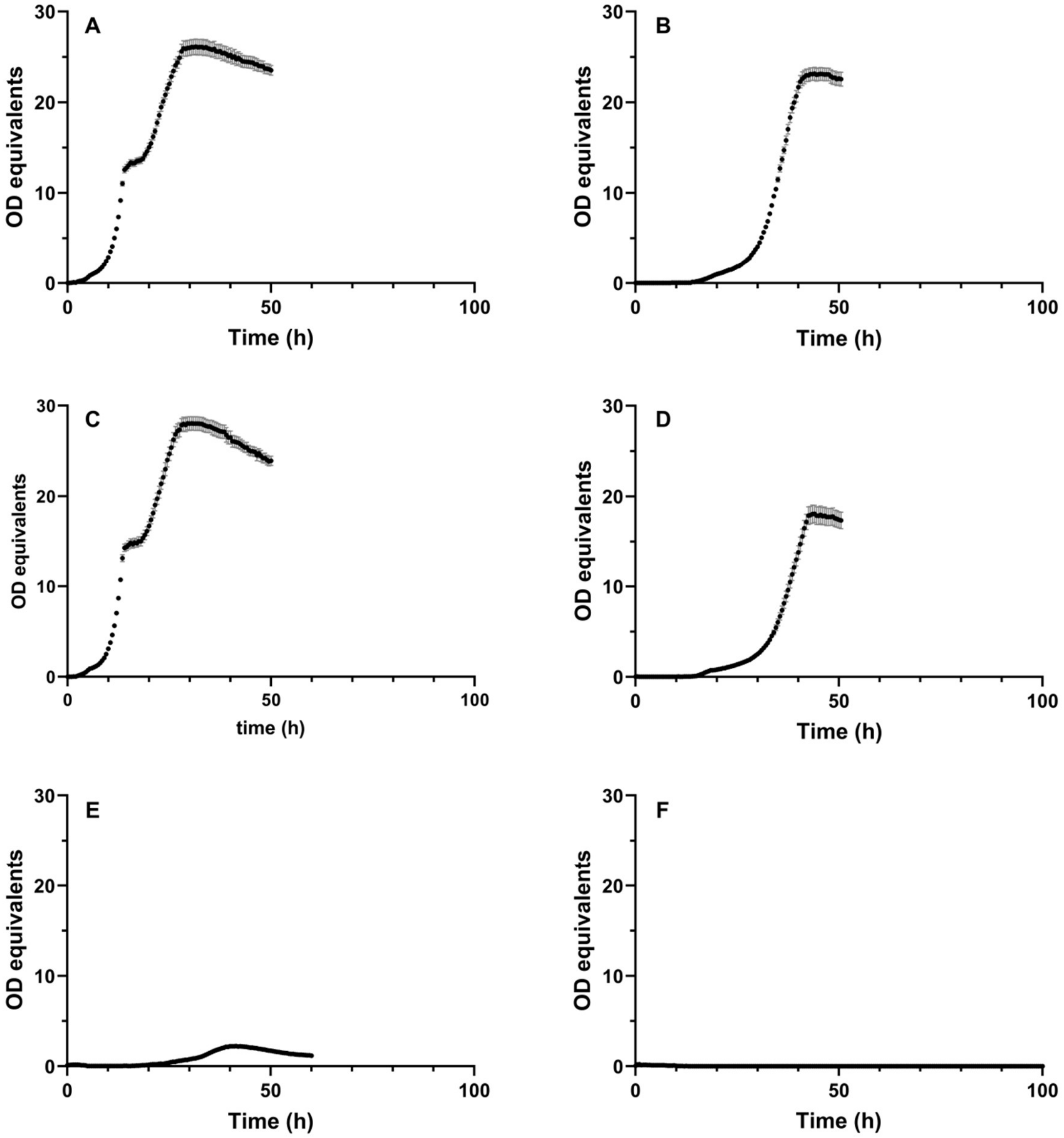

Figure S3: OD-equivalents obtained from Growth Profiler experiments. Strains IMX585 (**A** and **B**), IMX1438 (*sga1Δ::TtTHC1*; **C** and **D**) and IMK870 (*sga1Δ::TtTHC1 erg1Δ*; **E** and **F**) were grown in synthetic medium with urea as nitrogen source and either glucose (**A, C** and **E**) or a mixture of ethanol and glycerol (**B, D** and **F**) as carbon source. Green values were measured with time intervals of 30 min and converted to OD equivalents based on a pre-made calibration curve (see Methods).

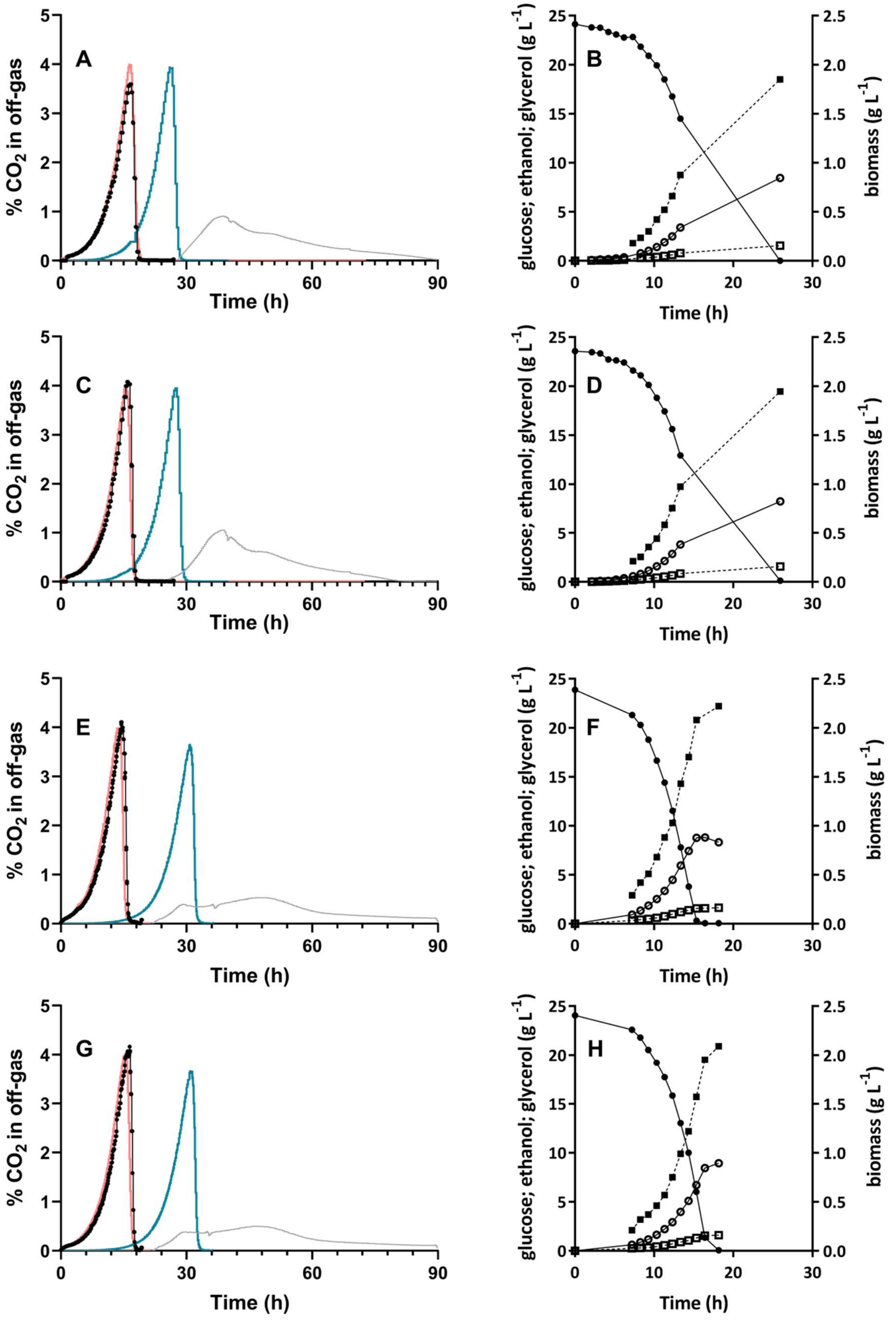

A
B
C
D
E
F
G
H
% CO2 in off-gas
Time (h)
glucose; ethanol; glycerol (g L-1)
biomass (g L-1)

Figure S4. **CO_2 production, metabolite and biomass profiles of anaerobic sequential batch cultures of *S. cerevisiae* IMX585 and IMX1438 with sterol supplementation.** All panels represent data from replicate anaerobic SBR experiments at 30°C on SMD-urea without pH control with reference strain IMX585 (panels A, B, C and D) and strain IMX1438 (*sga1Δ::TtTHC1;* panels E, F, G and H). **A, C, E and G**: Percentage of CO_2 in off-gas during the carry-over phase on medium without anaerobic growth factors (gray line) and the first (blue line), second (black line and dots) and third (red line) batch cycles on medium supplemented with Tween 80 and ergosterol. **B, D, F and H**: Measurement values of glucose (closed circles), biomass (closed squares), ethanol (open circles) and glycerol (open squares).

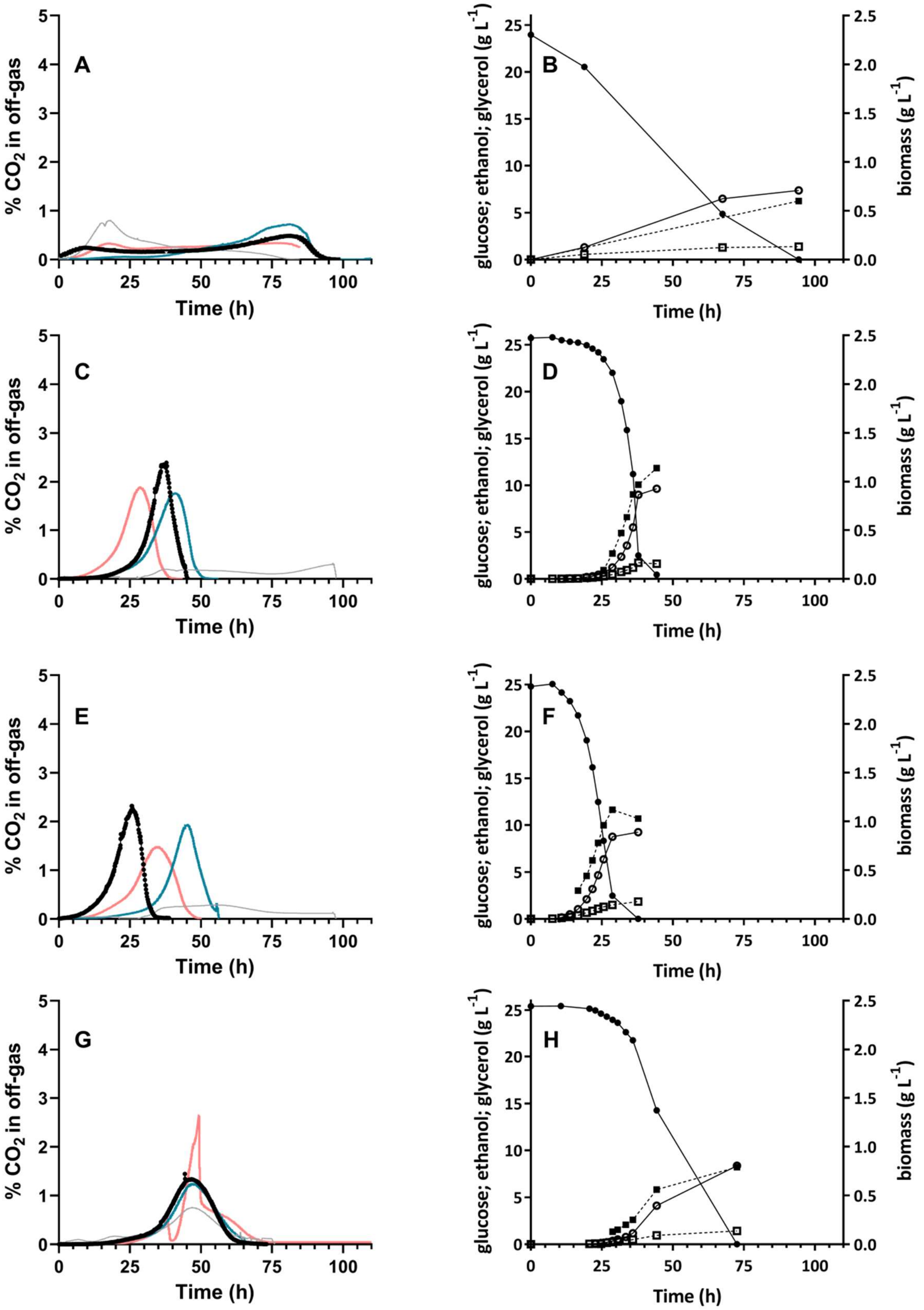

A
B
C
D
E
F
G
H
% CO_2 in off-gas
glucose; ethanol; glycerol (g L^{-1})
biomass (g L^{-1})
Time (h)

70

71

Figure S5. CO_2 production, metabolite and biomass profiles of anaerobic sequential batch cultures of *S. cerevisiae* IMX585, IMX1438 and IMK870 without sterol supplementation. All panels represent data from replicate anaerobic SBR experiments at 30°C on SMD-urea without pH control with reference strain IMX585 (panels A and B), strain IMX1438 (*sga1Δ::TtTHC1;* panels C, D, E and F) and strain IMK870 (*sga1Δ::TtTHC1 erg1Δ*; panels G and H). **A, C, E and G**: Percentage of CO_2 in off-gas during the carry-over phase on medium without anaerobic growth factors (gray line) and the first (blue line), second (black line and dots) and third (red line) batch cycles on medium supplemented with Tween 80 and ergosterol. **B, D, F and H**: Measurement values of glucose (closed circles), biomass (closed squares), ethanol (open circles) and glycerol (open squares).

Calculations S1. Dilution of ergosterol pool during bioreactor experiments

To perform the calculations presented below, the following assumptions were made. It was assumed that 1 g of dry biomass is equivalent to roughly 3.4×10^{10} cells. Furthermore, the ergosterol-content of biomass of CEN.PK113-7D was assumed to be approximately 1.5 mg (g biomass)$^{-1}$, when grown anaerobically in presence of exogenous ergosterol, as measured in anaerobic SBR experiments described in this work (Table S3). An optical density (660 nm) versus biomass dry weight ratio was calculated based on measurements from anaerobic SBR experiments to convert OD measurements ($0.134\times OD_{660} + 0.0201$ = biomass (g L^{-1})). Based on measurements of optical density at the start and end of each batch in sterol-free cultures of strain IMK870 (*sga1Δ::TtTHC1 erg1Δ*), an average of 4.76 generations per batch was assumed (Table S2).

With an initial OD_{660} of 0.2, the batches were started at a biomass concentration of approximately 0.046 g biomass L^{-1}. This leads to an estimated cell concentration of 1.56×10^{9} cells L^{-1}. The 0.046 g biomass would contain 0.069 mg ergosterol, and with a molecular weight of 396.66 g mol^{-1} for ergosterol and Avogadro's number (6.02214076×10^{23}), this would come down to roughly 6.7×10^{7} molecules $cell^{-1}$ at the start of the experiment. With an average number of 4.76 generations per batch, the number of molecules per cell at the end of each subsequent batch would be roughly 2.5×10^{6}, 9.1×10^{4}, 3.4×10^{3}, 124 and finally 4.6 molecules of ergosterol per cell, at the end of five subsequent batches, respectively.
